# Association between anemia in pregnancy with low birth weight and preterm birth in Ethiopia: A systematic review and meta-analysis

**DOI:** 10.1371/journal.pone.0310329

**Published:** 2024-09-12

**Authors:** Girma Beressa, Susan J. Whiting, Melesse Niguse Kuma, Bikila Lencha, Tefera Belachew

**Affiliations:** 1 Department of Public Health, Madda Walabu University, Goba, Ethiopia; 2 Nutrition and Dietetics Department, Faculty of Public Health, Jimma University, Jimma, Ethiopia; 3 College of Pharmacy and Nutrition, University of Saskatchewan, Saskatoon, Canada; Debre Markos University, ETHIOPIA

## Abstract

**Background:**

Anemia in pregnancy has been associated with a number of adverse birth outcomes, such as low birth weight (LBW) or preterm birth (PTB). However, the evidence from primary studies on anemia in pregnancy with LBW and PTB is contentious. Moreover, a systematic review and meta-analysis to summarize these findings have not been conducted for Ethiopia. This study aimed to synthesize the best available evidence and quantify the strength and direction of the association of anemia in Ethiopia.

**Methods:**

This review examined women with singleton pregnancies with low birth weight (LBW) and preterm birth (PTB). We retrieved studies from PubMed, Wiley, Cochrane databases, and Google Scholar from inception to February 2, 2024. The World Health Organization (WHO) defines anemia in pregnancy as a low blood haemoglobin (Hgb) concentration below 11 g/dl or a hematocrit level of < 33%. When the newborn’s weight was below 2500 g, LBW was considered. Preterm birth refers to the birth of a baby before 37 completed weeks of gestation. Meta-analysis was conducted using fixed and random effects models. The degree of heterogeneity, publication bias, and quality of the evidence of studies was assessed.

**Results:**

There were 35 and 8 studies, with 14,319 and 3,265 respondents included in the meta-analysis for LBW and PTB, respectively. Neonates born to women who had normal Hgb levels were less likely to be LBW [pooled odds ratio (POR) = 0.22, 95% CI: (0.17, 0.28); I^2^ = 80%] (low-quality evidence). Neonates born to women with normal Hgb levels had a lower risk of PTB [POR = 0.22, 95% CI: 0.18, 0.28; I^2^ = 19%] (very low-quality evidence). The effect size estimate remained significant after sub-group analysis based on study design and province, except in two retrospective cohort studies for LBW.

**Conclusion:**

The findings suggest major implications for strengthening the implementation of nutrition policies to prevent anemia during pregnancy in Ethiopia. Further research is warranted to assess interventions that are effective in combating maternal anemia to reduce rates of LBW and PTB.

## Introduction

Low birth weight (LBW), preterm birth (PTB), and stillbirth (fetal death at or after 28 gestational weeks) are among other adverse birth outcomes. These are still major public health issues worldwide, particularly in low and middle-income countries (LMICs) [1]. Low birth weight is defined by the World Health Organization (WHO) as a weight at birth of less than 2.5 kg (5.5 lb), irrespective of gestational age. Globally, the prevalence of LBW in 2015 was 14.6%. In 2015, an estimated 20.5 million live births were LBW, with 91% from LMICs, particularly Southern Asia (48%) and SSA (24%) [2].

Low birth weight is closely associated with fetal and neonatal mortality and morbidity, impaired growth and cognitive development, and chronic diseases later in life. Globally, an estimated 13.4 million newborn babies were born preterm (< 37 weeks) in 2020. The most significant gaps in national routine data for preterm births are found in Southern Asia and SSA, which also have the largest estimated preterm birth burden [3]. Ethiopia is one of the LMICs in SSA with high neonatal mortality [4]. In Ethiopia, the incidence of PTB ranges from 4.4 to 25.9% [5–9]. The pooled prevalence of PTB is 10.48% [10].

The WHO defines anemia in pregnancy as a low blood hemoglobin concentration, i.e., below 11 g/dl, or a hematocrit level of less than 33% [11]. Globally, anemia is one of the major public health concerns that affects 32.4 million (38.2%) pregnant women around the world [11, 12]. Worldwide, it has been reported that nearly 510,000 maternal deaths occur per year associated with childbirth or early post-partum. Approximately 20% of maternal deaths are caused by anemia, with the majority of deaths occurring in LMICs [13]. In Africa, prenatal anemia was detected in 48.7% of mothers [14]. Anemia during pregnancy has been reported to cause LBW, PTB, as well as perinatal, neonatal, and maternal mortality [15–21]. However, other studies report no association between maternal anemia with LBW or PTB [6, 22, 23], and, notably, these studies were conducted in SSA.

Nearly one-quarter of Ethiopian women in the reproductive age group are anemic and 29% of them are pregnant [24, 25]. The magnitude of prenatal anemia varies from 7.9 to 56.8% in Ethiopia [26–30]. A meta-analysis carried out in Ethiopia revealed that the pooled prevalence of anemia among pregnant women was 31.66% [31]. Those studies carried out in Ethiopia revealed that there was an association between anemia in pregnancy and LBW or PTB [32–41]. Nevertheless, the evidence from primary studies on anemia in pregnancy with LBW and PTB is equivocal. Therefore, this study aimed to synthesize the best available evidence and quantify the strength and direction of the association between anemia in pregnancy with low birth weight and preterm birth in Ethiopia.

### Review questions

Is there an association between anemia in pregnancy and low birth weight and preterm birth in Ethiopia?

## Methods

### Study design

This systematic review and meta-analysis were prepared using PRISMA reporting guidelines [42] (S1 Table). The systematic review was conducted following the Joanna Briggs Institute (JBI) methodology for systematic reviews of association evidence [43, 44]. The meta-analysis was prospectively registered in PROSPERO 2020: CRD42020207520 (available at https://www.crd.york.ac.uk/PR). The initial anticipated inception and completion times were updated.

### Eligibility criteria

#### Population

Studies of women where LBW and PTB data were provided were included in the review to determine the singleton pregnancy relationship with anemia in pregnancy and subsequent LBW and PTB. Multiple births were excluded, as were articles that were not full papers, reviews, qualitative studies, books, conferences, and proceedings, as well as animal studies.

#### Exposure of interest

Anemia in pregnancy was an exposure variable.

### Outcomes of interest

Low birth weight (LBW) and preterm birth (PTB) were the outcome variables. The gestational age (GA) was assessed using LMP and/or early ultrasound [45].

### Types of studies

The current review included observational studies (cross-sectional, case-control, retrospective, and prospective cohort studies) that reported an association between anemia or hemoglobin levels in pregnancy and subsequent LBW and PTB. This review considered all studies conducted in health facilities or community-based settings in Ethiopia.

### Search strategy

The search strategy aimed to locate both published and unpublished studies at the preprint stage that were written in English. The search was conducted from the inception of scientific databases until February 02, 2024. A three-step search strategy was utilized, with an initial search of PubMed undertaken for the analysis of text words, followed by a search using keywords and index terms across PubMed, Wiley Online Library, Cochrane Library, and Google Scholar, and eventually an examination of all articles retrieved for critical appraisal.

Various Boolean operators and terms were used to develop the search strategies. Specifically, to increase the comprehensiveness of the search results and fit the advanced PubMed database, medical subject headings (MeSH), key terms, and the search strategy were used. The search strategy was tailored to each scientific database to employ the appropriate search terms and available resources (S2 Table).

### Study selection

**Fig 1:**
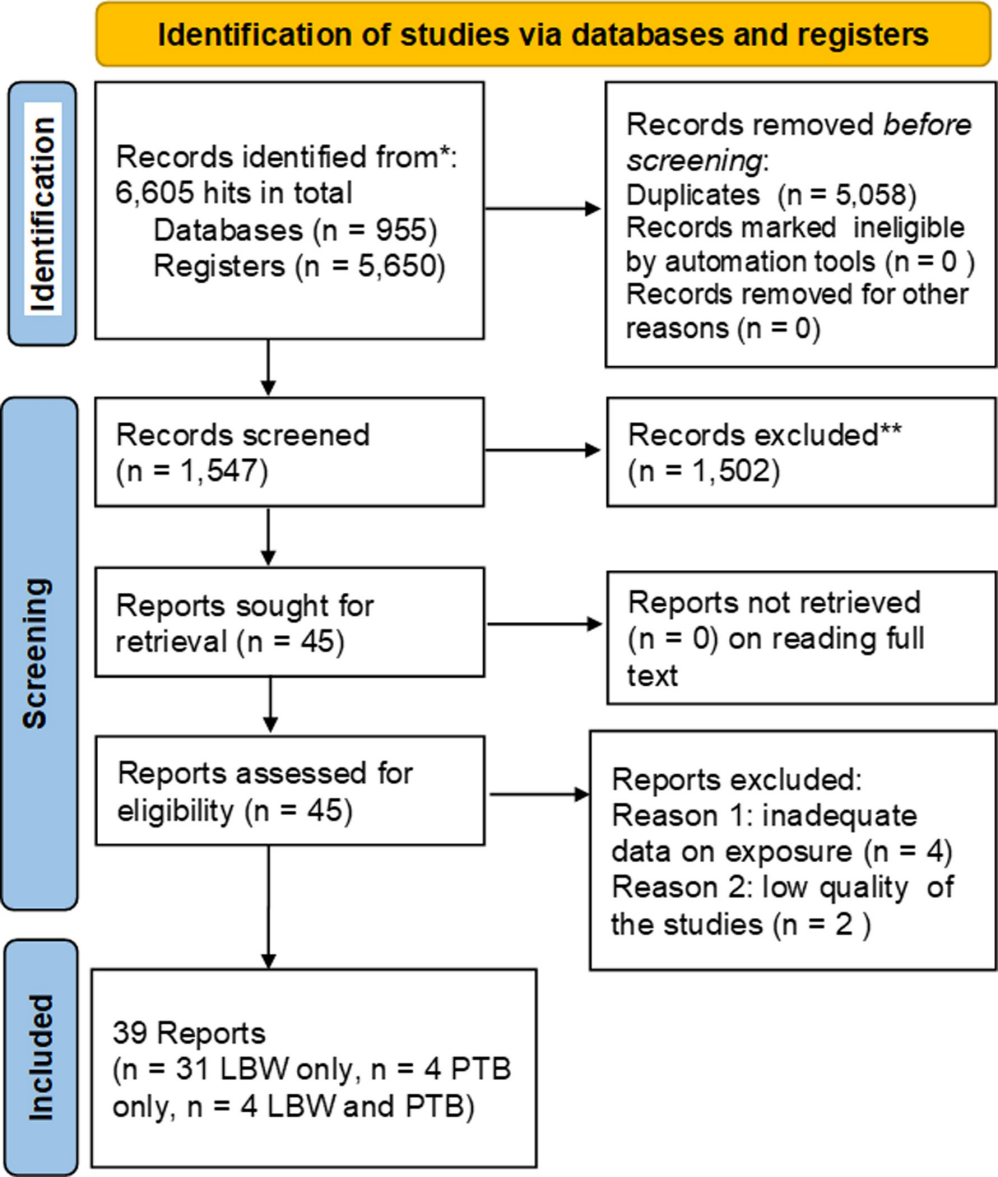
The study selection process. PRISMA 2020 flow diagram of association between anemia in pregnancy with low birth weight and preterm birth in Ethiopia, from inception to February 2, 2024.

Following the search, all identified citations were collated and uploaded into Endnote version X9 (Thomson Reuters, Philadelphia, PA, USA) software, and duplicates were removed. Two reviewers (GB and MNK) independently screened titles and abstracts against the inclusion criteria for the review. Potentially relevant studies were retrieved in full, and their citation details were imported into the JBI System for the Unified Management, Assessment, and Review of Information (JBI SUMARI) (JBI, Adelaide, Australia), https://www.jbisumari.org/). The full texts of selected citations were assessed in detail against the inclusion criteria by two or more independent reviewers. Reasons for the exclusion of papers in the full text that did not meet the inclusion criteria were recorded. Any disagreements that arose between the reviewers at each stage of the selection process were resolved through discussion, or with an additional reviewer/s. The results of the search and the study inclusion process were reported in full in the final systematic review and presented in the Preferred Reporting Items for Systematic Reviews and Meta-analyses (Fig 1 [46]).

### Missing data handling

We handled missing data by carefully considering the types of missing data and conducting sensitivity analyses.

### Assessment of methodological quality

Two independent reviewers critically appraised eligible studies at the study level using standardized critical appraisal instruments for cross-sectional studies, case-control studies, and cohort studies [44, 47] (S3 Table) and quality assessment (QA) (Tables 1–4 in S3 Table). Studies that did not meet a certain quality threshold were excluded, and reasons for their exclusion were provided in the (S4 Table). Any discord that arose between the reviewers was resolved through discussion or with a third reviewer. Studies were considered low-risk when they scored 50% or above on the quality of assessment indicators [48].

### Data extraction

Two independent reviewers (GB and MNK) extracted data from papers included in the review using the standardized data extraction tool for association available in JBI SUMARI software (available at https://www.jbisumari.org/) and the JBI manual for evidence synthesis [47]. The data extraction included specific details about the study: region, study type, sample size, outcomes measured, and main results for cross-sectional studies; for case-control studies: province or region, context, participant characteristics, sample size, exposures or variables measured, and main results; whereas for cohort studies: study, region, study type, participants, sample size, outcome assessed, and main results (Table 1). Any disagreements that arose between the reviewers were resolved through discussion or with a third reviewer.

**Table 1:** Characteristics of included studies in the systematic review and meta-analysis of the association between anemia in pregnancy with low birth weight (LBW) and preterm birth (PTB), Ethiopia, from inception to February 02, 2024.

Author/year	Region	Setting	Design	Sample size	Time of Hgb measures	Outcomes	Main description of results	URL	Name of data extractors	Date of data extraction
Halil et al., 2019 [37]	SNNP	Facility-based	Cross-sectional	363	during pregnancy	LBW	The prevalence of LBW was 12.7%. Not attending ANC visit, GA < 37 weeks, Hgb level ≤ 11g/dl, and a history of smoking were predictors of LBW.		GB and MNK	18/7/2021
Gebregzabiherher et al., 2017 [67]	Tigray	Hospital-based	Cross-sectional	424	during pregnancy	LBW	The prevalence of LBW was 10%. The predictors were mothers with normal Hgb, IFA, and HIV status.		GB and MNK	18/7/2021
Abera et al., 2019 [66]	SNNP	Facility-based	Cross-sectional	358	during pregnancy	LBW	The prevalence of LBW was 17.3%. Mothers having MUAC < 23 cm and with Hgb < 11 mg/dl have increased odds of delivering LBW.		GB and MNK	18/7/2021
Aboye et al., 2018 [39]	Tigray	institution based	Cross-sectional	308	during pregnancy	LBW	The prevalence of LBW was 8.8%. GA, ANC visit, anemia during pregnancy, and drinking alcohol were significantly associated with LBW.		GB and MNK	16/7/2021
Mekie et al.,2019 [40]	Amhara	Facility-based	Cross-sectional	28	during pregnancy	LBW	About 12.0% of LBW babies were delivered. The Hgb levels was significant predictors of LBW.		GB and MNK	16/7/2021
				2						
Kelkay et al., 2019 [92]	Tigray	institution	Cross-sectional	325	during pregnancy	PTB	The prevalence of singleton PTB was 16.9%. Hgb < 11gm/dl and history of giving LBW baby were statistically associated with singleton PTB.		GB and MNK	16/7/2021
Abdo et al., 2016 [68]	SNNP	Facility-based	Cross-sectional	327	during pregnancy	LBW, PTB, stillbirth	About 25.0% of women had adverse birth outcomes. Stillbirth, PTB, and LBW with the proportion of 8.6%, 8.6%, and 9.8%, respectively.		GB and MNK	16/7/2021
Cherie and Mebratu, 2017 [38]	Amhara	Hospital	Cross-sectional	462	during pregnancy	LBW, PTB, Stillbirth	The prevalence of adverse birth outcomes was 32.5%. Out of 462 births, 8.2% were stillbirth, 16.7% were LBW, 15.2% PTB and 8.4% were with visible birth defects.		GB and MNK	16/7/2021
Jember et al., 2020 [72]	Amhara	Institution	Cross-sectional	358	during pregnancy	LBW	The prevalence of LBW was 15.6%. Maternal age < 20 years and GA < 37 weeks were significantly associated with LBW.		GB and MNK	16/7/2021
Aynie et al., 2020 [34]	Amhara	Facility-based	Cross-sectional	292	during pregnancy	LBW	Having a previous history of LBW and having an Hgb level of < 11g/dl were statistically associated with LBW.		GB and MNK	16/7/2021
Chanie and Dilie., 2018 [32]	Amhara	Hospital	Cross-sectional	243	during pregnancy	LBW	The prevalence of LBW was 26.3%. Anemia was significantly associated with newborn birth weight.		GB and MNK	17/7/2021
Gudeta et al., 2019 [71]	SNNP	Hospital	Cross-sectional	1980	during pregnancy	LBW	The prevalence of LBW was 7.5%. Iron intake during pregnancy, induced labour, and GA were significantly associated with LBW.		GB and MNK	17/7/2021
Adane & Dachew, 2018 [69]	Amhara	Hospital	Cross-sectional	662	during pregnancy	LBW	The prevalence of LBW was 11.6%. Low income, GA < 37 weeks, MUAC 0 < 23 cm, and PIH were factors associated with LBW		GB and MNK	17/7/2021
Lemlem et al., 2021 [33]	Amhara	Institution	Cross-sectional	660	during pregnancy	LBW	The prevalence of LBW was 17.4%.		GB and MNK	17/7/2021
Ekubagewargies et al., 2019 [70]	Amhara	Institution	Cross-sectional	240	during pregnancy	LBW	The prevalence of LBW was 12.9%. No history of preeclampsia and being preterm were significantly associated with LBW.		GB and MNK	16/7/2021
Muhumad et al., 2021 [93]	Somali	Facility	Cross-sectional	607	during pregnancy	PTB	About 12.3% were PTB. Being a rural resident, PIH, and LBW of the newborn were significantly associated with PTB		GB and MNK	16/7/2021
Girma & Abebaw, 2018 [35]	Addis Ababa	Hospital	Cross-sectional	411	during pregnancy	LBW	The odds of LBW delivery among mothers with a previous history of stillbirth and LBW were about 4 and 12 times higher than those with no history, respectively. Similarly, mothers who delivered a PTB and those who were anemic were about 6 and 14 times higher than their counterparts, respectively.		GB and MNK	20/7/2021
Kure et al., 2021 [74]	Harari	Facility based	Cross-sectional	403	during pregnancy	LBW	The prevalence of LBW was 23.3%. Maternal anemia was statistically associated with LBW.		GB and MNK	20/7/2021
Kumlachew et al., 2018 [73]	Benishangul	Hospital	Cross-sectional	375	during pregnancy	LBW	The prevalence of LBW was 14.9%. Being anemia during pregnancy and lack of iron supplementation were predisposing factors to LBW.		GB and MNK	20/7/2021
Engidaw et al., 2022 [75]	Amhara	Hospital	Cross-sectional	211	during pregnancy	LBW	The prevalence of LBW among newborns was 26.0%. The independent effect of anaemia on LBW was 4.19.		GB and MNK	3/2/2024
Girma et al. 2019 [84]	Oromia	Facility Based	Case-control	93 Cases, 186 Controls	during pregnancy	LBW	No IFA, anemia, and inadequate minimum DDS of women were factors associated with LBW		GB and MNK	18/7/2021
Hailemichael et al., 2020 [85]	Tigray	Hospital-based	Case-control	135 Cases, 270 Controls	during pregnancy	LBW & PTB	Less than four ANC, not receiving dietary counselling, & < 11 g/dl Hgb level were significantly associated with adverse birth outcomes.		GB and MNK	18/7/2021
Hailu & Kebede, 2018 [79]	Amhara	Facility	Case-control	147 Cases, 249 Controls	during pregnancy	LBW	PTB, history of any physical trauma experienced during pregnancy, and history of any pregnancy complication were predictors of LBW.		GB and MNK	18/7/2021
Mohammed et al., 2021 [41]	SNNP	Hospital	Case-control	101 Cases, 303 Controls	during pregnancy	LBW	Mothers who did not receive IFAS during pregnancy, mothers who had anemia during pregnancy, and inadequate MDD-W were significant predictors of LBW.	https://www.researchsquare.com/article/rs-348265/v1	GB and MNK	16/7/2021
Baye Mulu et al., 2020 [76]	Addis Ababa	Institution	Case-control	Cases: 90; Controls:180	during pregnancy	LBW	Gestational HTN, incomplete ANC visit, and low maternal educational status predictors of LBW.		GB and MNK	16/7/2021
Sahlu et al., 2020 [82]	Addis Ababa	Institution	Case-control	Cases: 116, controls: 352 controls	during pregnancy	LBW	Mothers having food insecurity, MUAC, HTN, and early age association with LBW		GB and MNK	16/7/2021
Ahmed et al., 2018 [81]	Amhara	Facility	Case-control	Cases: 93, Controls: 186 controls	during pregnancy	LBW	The absence of IFAS, maternal anemia, and inadequate dietary diversity during the current pregnancy were significant determinants of LBW		GB and MNK	16/7/2021
Tilahun & Hailemarium, 2021[83]	SNNP	Hospital	Case-control	96 cases, 384 controls	during pregnancy	LBW	Not having IFAS during pregnancy, PTB, and history of pregnancy complications were determinants of LBW.	https://pdfs.semanticscholar.org/50fd/8aa911e5160add7d338d1017c408c0958284.pdf	GB and MNK	16/7/2021
Bekele et al., 2020 [77]	SNNP	Institution	Case-control	Cases: 118, Controls: 236	during pregnancy	LBW	The odds of PIH & not taking IFA during pregnancy were higher among mothers of the cases.		GB and MNK	16/7/2021
Wassie et al., 2020 [36]	Amhara	Institution	Case-control	Cases: 105 Controls: 209	during pregnancy	PTB	Lower level Hgb level was positively associated with PTB	https://www.researchsquare.com/article/rs-30092/v1	GB and MNK	16/7/2021
Gebrehawerya et al., 2018 [78]	Amhara	Facility	Case-control	Cases: 96, Controls: 191	during pregnancy	LBW	About 79.2% of cases and 93.2% of controls had ANC at least once. Parity, ≤ ANC visits, anemia, and PIH were significantly associated with LBW.		GB and MNK	16/7/2021
Nebi et al., 2019 [80]	Oromia	Facility	Case-control	Cases: 108, Controls: 210	during pregnancy	LBW	Being rural with, parity ≥ 2, history of hypertension, and maternal MUAC < 21cm were statistically significant.	https://www.researchsquare.com/article/rs-4025/v1	GB and MNK	16/7/2021
Tadese et al., 2021 [87]	Addis Ababa	Facility	Unmatched case control	453 (151 cases and 302 controls)	during pregnancy	LBW	Maternal weight during pregnancy were significant determinants of LBW.		GB and MNK	3/2/2024
Seid et al., 2022 [86]	SNNP	Facility	Unmatched case control	84 cases and 168 controls	during pregnancy	LBW	Mothers who did not receive IFAS during pregnancy and maternal Hgb levels were determinants of LBW.		GB and MNK	3/2/2024
Desta et al., 2019 [88]	SNNP	Hospital	Retrospective cohort	70, Exposed, LBW < 2.5 kg 350, Non exposed (NBW)	during pregnancy	LBW	The incidence of LBW was 16.6%. LBW newborns were associated with a low APGAR score and early newborn death.		GB and MNK	16/7/2021
Zenebe et al., 2020 [89]	SNNP	Hospital	Retrospective cohort	277 HIV-ve, 252 HIV+ve	during pregnancy	LBW	The prevalence of LBW was also significantly higher in the HIV-exposed group (22.2%).		GB and MNK	16/7/2021
Brhane et al., 2019 [94]	Tigray	Facility	Prospective cohort	153 exposed mothers/307 unexposed mothers	during pregnancy	PTB	The total incidence of PTB was 10.4%.		GB and MNK	19/7/2021
Zerfu et al., 2016[90]	Oromia	Facility	Prospective cohort	34 LBW, 51 PTB, 17 stillbirth	during pregnancy	LBW, PTB, stillbirth	One in five (19.8%) experienced at least one of the APO: 34 (9.1%) gave birth to LBW babies, 51(13.6%) had PTB, and 17 (4.5%) experienced stillbirth. Being nonanemic at term was associated with lower APO risks		GB and MNK	19/7/2021
Fite et al., 2022 [91]	Oromia	Community	Prospective cohort	412	during pregnancy	LBW	About 20.2% of newborns were born with LBW. The prevalence of LBW was 5 times higher among women who were iron deficient during pregnancy.		GB and MNK	3/2/2024

ANC: antenatal care; APO: adverse pregnancy outcomes; DDS: dietary diversity score; GA: gestational age; Hgb: hemoglobin; IFAS: iron folic acid supplement; HTN: hypertension; LBW: low birth weight; MUAC: mid-upper arm circumference; MDD-W: minimum dietary diversity for women; NA: Not applicable; PTB: preterm birth; PIH: pregnancy-induced hypertension; SNNPR: Southern Nations, Nationalities and Peoples’ Region

### Data synthesis and analysis

Where possible, quantitative data were pooled in a statistical meta-analysis using Review Manager (RevMan) version 5.3 and STATA software version 14. A DerSimonian and Laird’s random and fixed effects model [49, 50] using the Mantel-Haenszel method was used to evaluate the significance of the results for LBW and PTB. The effect size was expressed as an odds ratio (OR) along with 95% confidence intervals (CI) around the summary estimate for dichotomous outcomes (LBW and PTB). Heterogeneity was assessed statistically using Tau^2^, the standard chi-squared (Cochran Q test), and Higgins I^2^ (I squared) tests. The conservative significance threshold of a p-value of < 0.1 for the Cochrane’s Q test was used to determine heterogeneity [51]. The I^2^ test statistics of 25%, 50%, and 75% were declared as low, moderate, and high heterogeneity, respectively [52–55]. Higgins’ I^2^ test statistic describes the percentage of variability in point estimates that is due to heterogeneity rather than sampling error [54]. Evidence of publication bias was also assessed visually by inspecting the funnel plot [56, 57] and more objectively using Harbord’s test at a 5% level of significance [50, 58]. A p-value of < 0.05 was used to declare statistical significance. The Duval and Tweedie nonparametric trim and fill analysis [59] was not performed to deal with publication bias, as there was no evidence of a risk of publication bias among the included studies. The Stata commands ’metabias’ and ’metaninf’ were used to deal with publication bias and sensitivity analysis, respectively. We prudently carried out subgroup analysis based on study design (cross-sectional, case-control, retrospective, and prospective cohort) and regions.

Sensitivity analyses using the leave-one-out approach were used to assess the robustness of the study results [60]. The findings of the meta-analysis were displayed on a forest plot. The quality of evidence for studies was assessed using the Grading of recommendation, assessment, development, and evaluation (GRADE) pro guideline development tool (GDT) software version 3.6.1 developed by the (GRADE) working group [61]. The GRADE system rates the quality of evidence as varying from high, moderate, low, and very low in five domains: risk of bias (RoB), inconsistency, indirectness, imprecision, and publication bias [62, 63].

### Ethical approval and consent to participate

Ethical approval is not applicable as this is not a primary study.

## Results

### Description of studies

The database search yielded a total of 6,605 records (Fig 1). After the removal of duplicates, 1,547 potentially relevant papers were retained for further review. After screening titles and abstracts, 45 papers were retained for full-paper examination. Of these, six full-text papers were excluded as they did not meet the inclusion criteria (i.e., two papers were excluded because they did not relate to outcome variables; four papers due to inadequate information on exposure variables; and two papers because of the low quality of the studies). A total of 45 papers were retained for methodological quality assessment and were critically appraised by the independent reviewers (S3 Table) and QA-Tables 1–4 in S3 Table. Subsequently, two papers [64, 65] were excluded after critical appraisal. Finally, thirty-five and eight studies contained 14,319 and 3,265 study subjects whose pregnancies were analysed for LBW and PTB, respectively, that were retained for meta-analysis (Fig 1). The included studies in the meta-analyses were eighteen cross-sectional studies [32–35, 37–40, 66–75], thirteen case-control studies [41, 76–87], two retrospective cohort studies [88, 89], and two prospective cohort studies [90, 91] that reported LBW. Many studies that described LBW were from Ethiopian regions: twelve Amhara studies [32–34, 38, 40, 69, 70, 72, 75, 78, 79, 81], ten Southern Nations, Nationalities, and Peoples’ Region (SNNPR) studies [37, 41, 66, 68, 71, 77, 83, 86, 88, 89], four Addis Ababa studies [35, 76, 82, 87], four Oromia studies [80, 84, 90, 91], three Tigray studies [39, 67, 85], one Harari study [74], and one Benishangul-Gumuz study [73]. Four cross-sectional studies [38, 68, 92, 93], two case-control studies [36, 85], and two prospective cohort studies [90, 94] were analysed for PTB, and there were two studies [36, 38] from Amhara, one study [68] from SNNPR, one study [90] from Oromia, three studies [85, 92, 94] from Tigray, and one study [93] from the Somali region, Ethiopia, and all reported PTB (Table 1).

### Methodological quality analysis

We endeavoured to include studies that fulfilled a high methodological quality standard by requiring that they meet at least 50% of the particular requirements for each study design checklist out of 100. Based on the results of the JBI-MAStARI assessment tool critical appraisal, the methodological quality of the included studies had a low RoB. However, two prospective cohort studies were excluded because of poor methodological quality.

For cross-sectional studies, some authors did not provide clear inclusion and exclusion criteria before the recruitment of study subjects [34, 39, 68, 72, 73]. The authors did not also describe the method of measuring exposures [39]. Besides, the authors did not use standard criteria for the measurement of the condition [68]. Moreover, the authors did not measure the outcome validly and reliably [33, 39, 68, 69, 93] (S3 Table) and QA-Table 1 in S3 Table.

For cases and controls, when the authors did not assess the exposure in a standard, valid, and reliable way or did not assess the outcomes in a standard, valid, and reliable way, the study was excluded [83] (S3 Table) and QA-Table 2 in S3 Table.

For cohort studies, some authors did not state strategies to address incomplete follow-up [94] (S3 Table) and QA-Table 3 in S3 Table. Nevertheless, two prospective cohort studies [64, 65] were excluded from the meta-analysis as they did not meet the minimal inclusion criteria. The studies were deemed to have insufficient information concerning the exposure of interest. Moreover, the reasons for the failure to follow up were not described and explored clearly. Furthermore, strategies to address incomplete follow-up were not delineated (S3 Table) and QA-Table 4 in S3 Table.

## Meta-analysis

### Relationship between anemia in pregnancy and low birth weight

**Fig 2:**
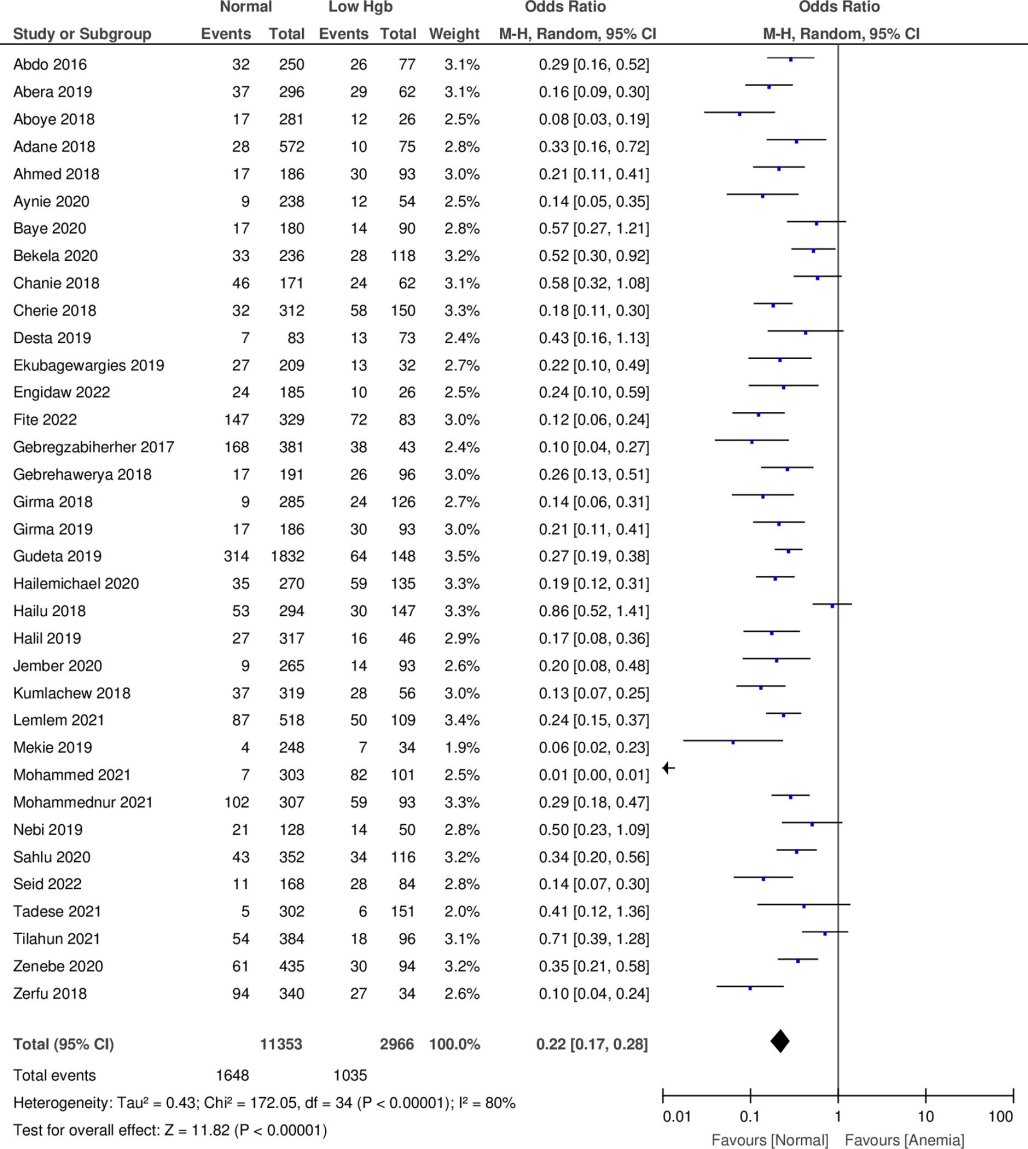
A forest plot of a meta-analysis of the association between anemia in pregnancy and the subsequent low birth weight of the neonate, Ethiopia.

The meta-analysis results of 35 studies revealed that neonates born to women who had normal Hgb levels were less likely to have LBW [Pooled odds ratio (POR) = 0.22, 95% CI: (0.17 to 0.28)]. The I^2^ statistic revealed that there was statistical evidence of heterogeneity among studies, and the heterogeneity was statistically significant (I^2^ = 80%, P < 0.00001) (**Fig 2**).

### Publication bias assessment

The risk of publication bias was assessed using a visual inspection of funnel plots. The funnel plots appeared substantially asymmetrical (i.e., tilted to the left side) (S1 Fig). However, Harbord’s test (P-value = 0.06) revealed that there was no statistical evidence of publication bias.

### Subgroup analysis for low birth weight

#### Subgroup analysis based on study design

The present subgroup analysis of 18 cross-sectional studies indicated that the odds of neonates born to women who had normal Hgb levels were [POR = 0.20, 95% CI: (0.16 to 0.25)] less likely to have LBW. The I^2^ test of a meta-analysis of eighteen cross-sectional studies indicated that there was heterogeneity among studies, and the heterogeneity was statistically significant (I^2^ = 48%, P = 0.01).

Similarly, there were 13 case-control studies in which there was a relationship between anemia in pregnancy and LBW [POR = 0.26, 95% CI: (0.15 to 0.47)]. The I^2^ test of a meta-analysis revealed that there was high heterogeneity, and the heterogeneity was statistically significant (I^2^ = 90%, P < 0.00001).

Likewise, there were two prospective cohort studies in which there was a relationship between anemia in pregnancy and LBW [POR = 0.11, 95% CI: (0.07 to 0.19)]. The I^2^ test of a meta-analysis revealed that there was no heterogeneity, and the heterogeneity was not statistically significant (I^2^ = 0%, P = 0.69). Nevertheless, there were two retrospective cohort studies in which there was no relationship between anemia during pregnancy and LBW [POR = 0.57, 95% CI: (0.20 to 1.66)], I^2^ **=** 80% (P = 0.02).

The overall effect estimates of the meta-analysis indicated that the odds of infants born to women who had normal Hgb levels were less likely to have LBW [(POR = 0.23, 95% CI: (0.18 to 0.29)]. The I^2^ test statistic of the overall effect estimates of the eighteen cross-sectional studies, thirteen case-control studies, two retrospective cohort studies [88, 89], and two prospective cohort studies [90] indicated that there was statistical evidence of heterogeneity among studies and the heterogeneity was statistically significant (I^2^ = 81%, P < 0.00001) (S2 Fig).

### Subgroup analysis based on study province

The subgroup analysis of the current twelve studies revealed that infants born to women who had normal Hgb levels were less likely to be LBW [POR = 0.26, 95% CI: (0.18 to 0.37)], (I^2^ = 71%, P < 0.0001) in Amhara, [POR = 0.22, 95% CI: (0.12 to 0.43)], (I^2^ = 92%, P < 0.00001) in SNNPR region, [POR = 0.32, 95% CI: (0.18 to 0.56)], (I^2^ = 54%, P = 0.09) in Addis Ababa city, [POR = 0.19, 95% CI: (0.10 to 0.38)], (I^2^ = 70%, P = 0.02) in Oromia, [AOR = 0.13, 95% CI: (0.07 to 0.23)], I^2^ = 46% (P = 0.16) in Tigray, [POR = 0.29, 95% CI: (0.18 to 0.47)] in Harari region, and [POR = 0.13, 95% CI: (0.07 to 0.25)] in Benishangul-Gumuz region. The overall effect sizes of the included studies by regions were [POR = 0.23, 95% CI: (0.18 to 0.29)], I² = 81% (P < 0.00001) (S3 Fig).

### Relationship between anemia in pregnancy and preterm birth

**Fig 3:**
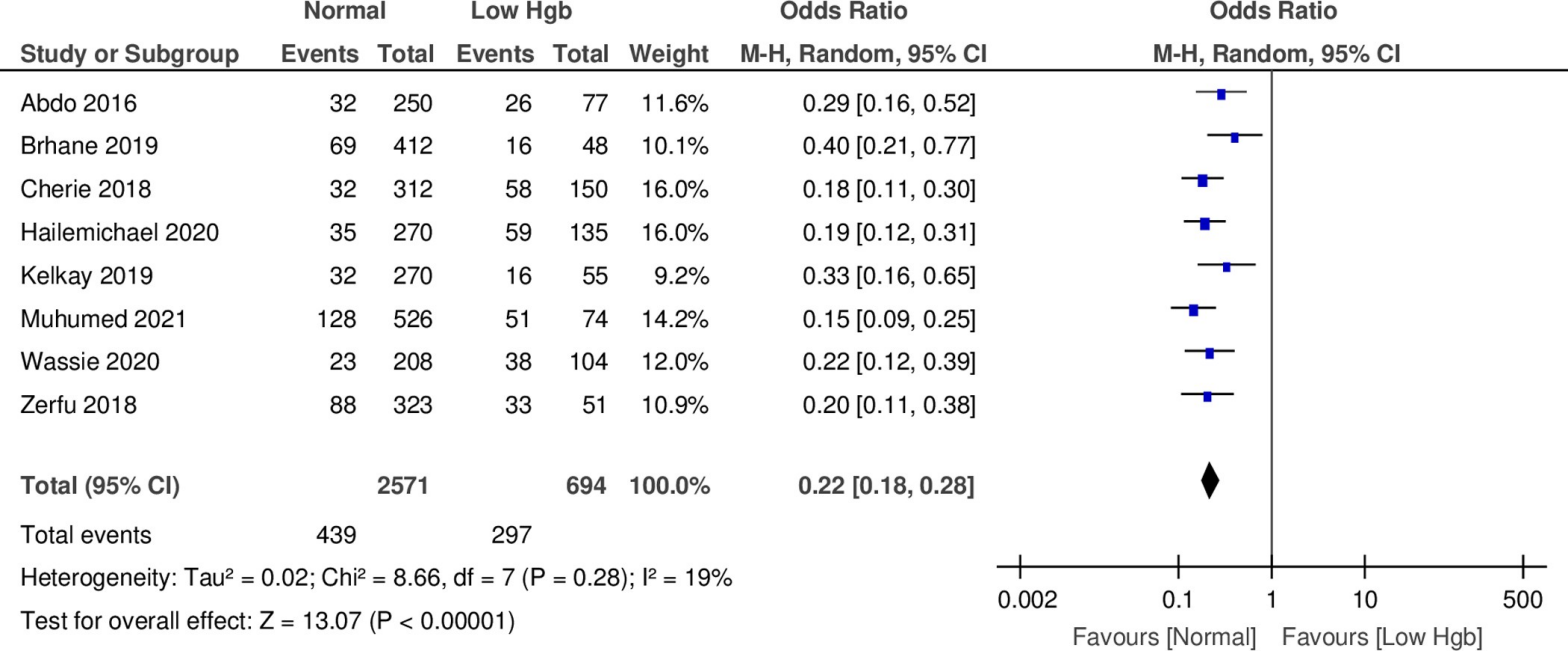
Forest plot for a meta-analysis of the association between anemia in pregnancy and subsequent preterm birth, Ethiopia.

The present meta-analysis findings of eight studies indicated that the odds of neonates born to women who had normal Hgb levels were less likely to have PTB [POR = 0.22, 95% CI: (0.18, 0.28)]. The I^2^ test indicated that there was low heterogeneity among studies, and the heterogeneity was statistically significant (I^2^ = 19%, P = 0.28) (**Fig 3**).

### Subgroup analysis based on study design

The sub-group analysis employing the fixed effect model of four cross-sectional studies revealed that the neonates born to women who had normal Hgb levels were less likely to have PTB [POR = 0.21, 95% CI: (0.16 to 0.27)]. The I^2^ test indicated that there was low heterogeneity among studies, and the heterogeneity was statistically significant (I^2^ = 39%, P = 0.18). Similarly, a sub-group analysis of two case-control studies indicated that the neonates born to women who had normal Hgb levels were less likely to have PTB [POR = 0.20, 95% CI: (0.14 to 0.29)]. Nonetheless, the I^2^ test indicated that there was no heterogeneity among studies, and that the heterogeneity was not statistically significant (I^2^ = 0%, P = 0.76).

A sub-group analysis of two prospective cohort studies revealed that the neonates born to women who had normal Hgb levels were less likely to have PTB [POR = 0.28, 95% CI: (0.18 to 0.43)]. The I^2^ test indicated that there was high heterogeneity among studies, and the heterogeneity was not statistically significant (I^2^ = 19%, P = 0.14). The overall effect sizes of the four cross-sectional, two case-control, and two prospective cohorts of the included studies were [POR = 0.22, 95% CI: (0.18 to 0.26)]. The I^2^ test indicated that there was low heterogeneity among studies, and the heterogeneity was not statistically significant (I^2^ = 19%, P = 0.28) (S4 Fig).

### Subgroup analysis based on study province

The sub-group analysis of the fixed effect model studies revealed that the neonates born to women who had normal Hgb levels were less likely to have PTB [POR = 0.19, 95% CI: (0.13, 0.28)], (I^2^ = 0%, P = 66) in Amhara, [POR = 0.29, 95% CI: (0.16 to 0.52)] in SNNPR region, and [POR = 0.26, 95% CI: (0.19 to 0.37)], (I^2^ = 44%, P = 0.17) in Tigray region, whereas the overall effect sizes of the included studies by regions were [POR = 0.22, 95% CI: (0.18 to 0.26)], I² = 19%; (P = 0.28) (S5 Fig).

### Sensitivity analysis of the included studies for anemia in pregnancy with low birth weight and preterm birth of neonates, Ethiopia

To assess the robustness of the study results, we meticulously performed sensitivity analyses using the leave-one-out approach. Nonetheless, there was no change in the overall (pooled) effect estimate (S6 and S7 Figs).

### Quality of evidence

The quality of the evidence in the included studies for LBW and PTB was rated as low and very low, respectively. The major reasons for downgrading the quality of evidence might be RoB and high heterogeneity among included studies, as well as the relatively smaller sample size (Table 2).

**Table 2:** Summary of findings (SoF) of the association between anemia in pregnancy with low birth weight and preterm birth, Ethiopia, from inception to February 02, 2024.

Outcomes	Relative effect (95% CI)	No. of studies	Study design	Risk of bias	Inconsistency	indirectness	imprecision	Publication bias	GRADE quality
LBW	**OR 0.22** (0.17 to 0.28)	31 studies	18 Cross-sectional, 13 case control & 2 retrospective and 2 prospective cohort studies	-1	-1	0	0	0	⊕⊕⊝⊝ **low**^1^^,^^2^^,^^3^^,^^4^^,^^5^
PTB	**OR 0.36** (0.31 to 0.41)	8 studies	4 Cross-sectional, 2 case control & 2 prospective cohort studies)^1^	-1	-1	0	-1	0	⊕⊝⊝⊝ **very low**^1^^,^^6^^,^^7^^,^^8^^,^^9^

The symbols + +‐‐ show the quality of the evidence

Abbreviations: CI, confidence interval; GRADE, grades of recommendation, assessment, development, and evaluation; LBW, low birth weight; OR, odds ratio; RR: relative risk; PTB, preterm birth

^1^ Downgraded one level as there is a serious risk of bias.

^2^Downgraded one as I^2^ was 80% and heterogeneity was present.

^3^Increase confidence one-level as the number of included studies was > 20 and imprecision and publication bias were considerable.

^4^Increase confidence as the large effect measure was RR < 0.5

^5^The meta-analysis revealed that there was a statistically significant association between Hgb and LBW.

^6^ Downgraded as I^2^ was 19% and heterogeneity was present.

^7^Downgraded as the sample size was not large enough.

^8^ Increase confidence due to the large effect measure i.e., RR < 0.5.

^9^ Increase confidence, as there was a negative association between anemia and PTB.

## Discussion

Our review identified that newborns born to women with anemia during pregnancy had a higher risk of LBW and PTB. The present finding that neonates born to women who had normal Hgb levels were 78% less likely to have LBW is in agreement with other systematic reviews and meta-analyses carried out in other countries [19, 21, 95–99] and [100]. However, several published meta-analyses showed either no effect of maternal anemia during pregnancy on LBW [101] or that high maternal Hgb was not significantly associated with LBW [21]. The possible reasons for differences in results could be because of variation in the socio-demographic characteristics of study subjects.

The present sub-group analyses of 18 cross-sectional studies revealed that the odds of neonates born to women who had normal Hgb levels were 80% less likely to be LBW. Likewise, the subgroup analysis of 13 case-control studies indicated that the odds of neonates born to women who had normal Hgb levels were 74% less likely to be LBW. This is in agreement with other studies [19] and [95]. However, the sub-group meta-analysis results of two retrospective cohort studies indicated that there was no relationship between normal Hgb during pregnancy and LBW, which is different from a meta-analysis [19]. The possible explanation might be because of the low power to detect the association using this design.

The overall effect estimates of the meta-analysis based on study designs indicated that the odds of infants born to women who had normal Hgb levels were 78% less likely to be LBW. The I^2^ statistic test of the overall effect estimates of the 18 cross-sectional, 13 case-control, two retrospective, and two prospective cohort studies indicated that there was statistical evidence of heterogeneity among studies and that the heterogeneity was statistically significant. The high heterogeneity among included studies could be attributed to differences in study subjects’ characteristics such as health, socioeconomic status, and nutritional status [102, 103].

The current systematic review and meta-analysis identified eight studies in which the odds of neonates born to women who had normal Hgb levels were 78% less likely to be PTB. This study’s findings agreed with those of other studies [95, 96, 101]. Likewise, the present result supports the WHO anemia policy brief [104]. The possible explanation might be because of the variety of study settings with low access to quality health care and the time difference between the reviews, in which the current review also included recent studies among study subjects. Moreover, this finding agreed with other meta-analyses [100] conducted in LMICs, which revealed that anemic women are associated with increased odds of giving PTB. This could be because anemia impairs oxygen transportation, resulting in placental insufficiency, which will ultimately result in PTB. However, the current study’s result is not in agreement with a meta-analysis carried out on HICs [20]. This is not unexpected, as in these countries there is likely to be good access to quality health care.

The mechanisms underlying the link between low maternal haemoglobin and birth outcomes are complex and multifactorial, and may include nutritional deficiencies (e.g., iron, vitamin A, folic acid, or vitamin B12 deficiency), infectious causes (e.g., malaria, schistosomiasis, hookworm infection, HIV), hemoglobinopathies (sickle cell anaemia, thalassemia), and inflammation [105]. Iron deficiency has been linked to up to 75% of all kinds of anaemia during pregnancy [105]. Iron deficiency is caused by inadequate food intake combined with increased systemic demand, poor absorption, or blood loss. Iron deficiency varies by geography, with a higher frequency in low-income nations. Iron needs and absorption vary throughout pregnancy, with lower requirements in the first trimester and a roughly three-fold rise in the third trimester due to increased maternal red blood cell mass expansion, placental demand, and foetal development [106]. Iron deficiency anemia (IDA) is related to reduced oxygen supply to the tissues, weariness, an increased risk of infection, and heart failure in severe cases [107]. Iron deficiency is caused by inadequate food intake combined with increased systemic demand, poor absorption, or blood loss. Iron deficiency varies by geography, with a higher frequency in low-income countries.

Iron needs and absorption vary throughout pregnancy, with lower requirements in the first trimester and a roughly three-fold rise in the third trimester due to increased maternal red blood cell mass expansion, placental demand, and foetal development [106]. IDA is related to reduced oxygen supply to the tissues, weariness, an increased risk of infection, and heart failure in severe cases [107]. IDA in children is linked to poor prenatal outcomes such as LBW and PTB. Although iron insufficiency has been generally linked to dietary factors (e.g., low iron intake or poor iron absorption), numerous non-nutritional causes should be considered as well. Inflammation (due to viral causes or low-grade inflammation exhibited in overweight or obese individuals) may potentially affect iron absorption and metabolism by increasing hepcidin levels, resulting in anemia of inflammation despite adequate iron reserves [106].

### Strengths and limitations of the review

The strengths of this review were its comprehensive search strategy and having at least two reviewers participate in each step of the review process. The heterogeneity and publication bias, as well as the quality of evidence for each outcome, were assessed. This systematic review and meta-analysis were based on studies carried out in Ethiopia, but a limitation is that no data was found in the three provinces. We found high heterogeneity within the included studies. Further, the search strategy was limited to studies published in the English language and could be subject to reporting bias. As the included studies were observational (cross-sectional, case-control, and cohort study designs), the outcome of interest might be affected by other confounding variables, such as sample size, dwelling, and study year, so the findings could not establish cause-and-effect relationships. Not all studies accounted for altitude when assessing hemoglobin levels. Some measurements for Hgb level were taken after delivery, and this may not be indicative of anemia in pregnancy and LBW and PTB.

## Conclusion

Neonates born to women who had normal hemoglobin (Hgb) levels were less likely to be LBW or PTB. The quality of evidence for studies was rated low to very low. Our findings highlight the importance of locally appropriate, priority interventions to improve maternal Hgb status during pregnancy to reduce the risk of LBW and PTB. Investing in maternal Hgb status is also a key strategy to diminish LBW and PTB. Therefore, to prevent maternal anemia, pregnant women are counselled to get ferrous folate supplements and iron-rich diets. Moreover, deworming pregnant women in the third trimester is crucial for the prevention and control of anemia.

## Recommendations for practice and research

It is imperative to identify which interventions are promoted to address maternal anemia, LBW, and PTB. Low-quality evidence indicates optimal nutrition intervention during which enhancing Hgb levels during pregnancy is effective in decreasing LBW. Very low-quality evidence reveals that investing in maternal Hgb status would be more effective in decreasing PTB. Interpretation should be given due attention because of methodological quality and high heterogeneity (high variations between and within studies). Further research, such as randomized control trials, is needed to establish strong evidence or ensure effective evidence-based food and nutrition policy.

## Supporting information

S1 Fig Funnel plot LBW. (TIF)

S2 Fig Forest plot study design LBW. (TIF)

S3 Fig Forest plot study province LBW. (TIF)

S4 Fig Forest plot study design PTB. (TIF)

S5 Fig Forest plot study province PTB. (TIF)

S6 Fig Sensitivity analysis LBW. (TIF)

S7 Fig Sensitivity analysis PTB. (TIF)

S1 Table PRISMA checklist. (DOCX)

S2 Table Search strategy. (DOCX)

S3 Table QA-Tables 1–4. (DOCX)

S4 Table Reasons for excluded studies. (DOCX)

## Abbreviations or acronyms

Hgb: Hemoglobin
LBW: Low Birth Weight
PTB: Preterm birth
