# Supplementary figures and images for: Association between anemia in pregnancy with low birth weight and preterm birth in Ethiopia: A systematic review and meta-analysis
Source: PLoS One. 2024 Sep 12;19(9):e0310329. doi: 10.1371/journal.pone.0310329 (PMC11392424; doi:10.1371/journal.pone.0310329)

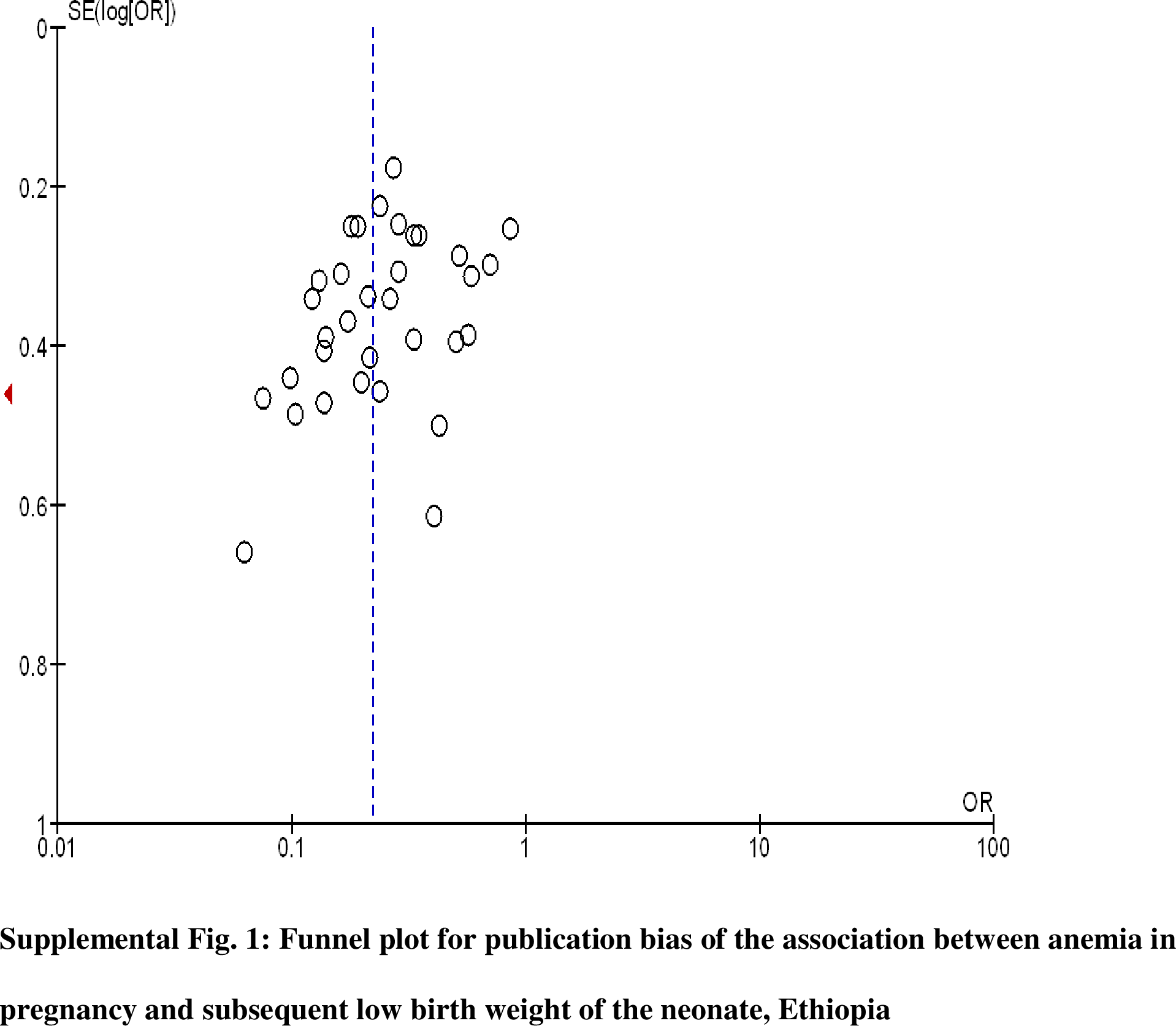

Supplement: S1 Fig — (TIF) [file pone.0310329.s001.tif]

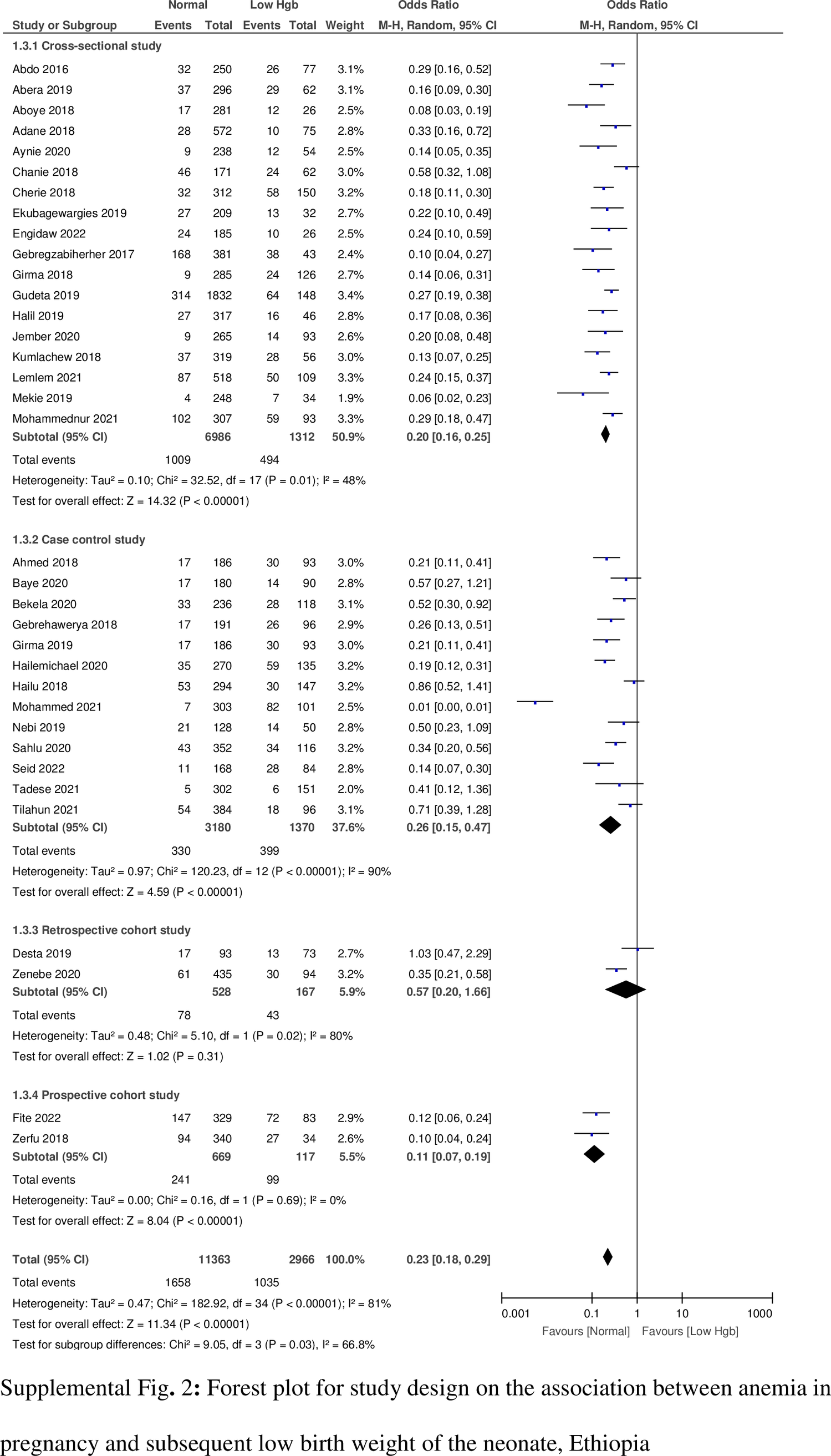

Supplement: S2 Fig — (TIF) [file pone.0310329.s002.tif]

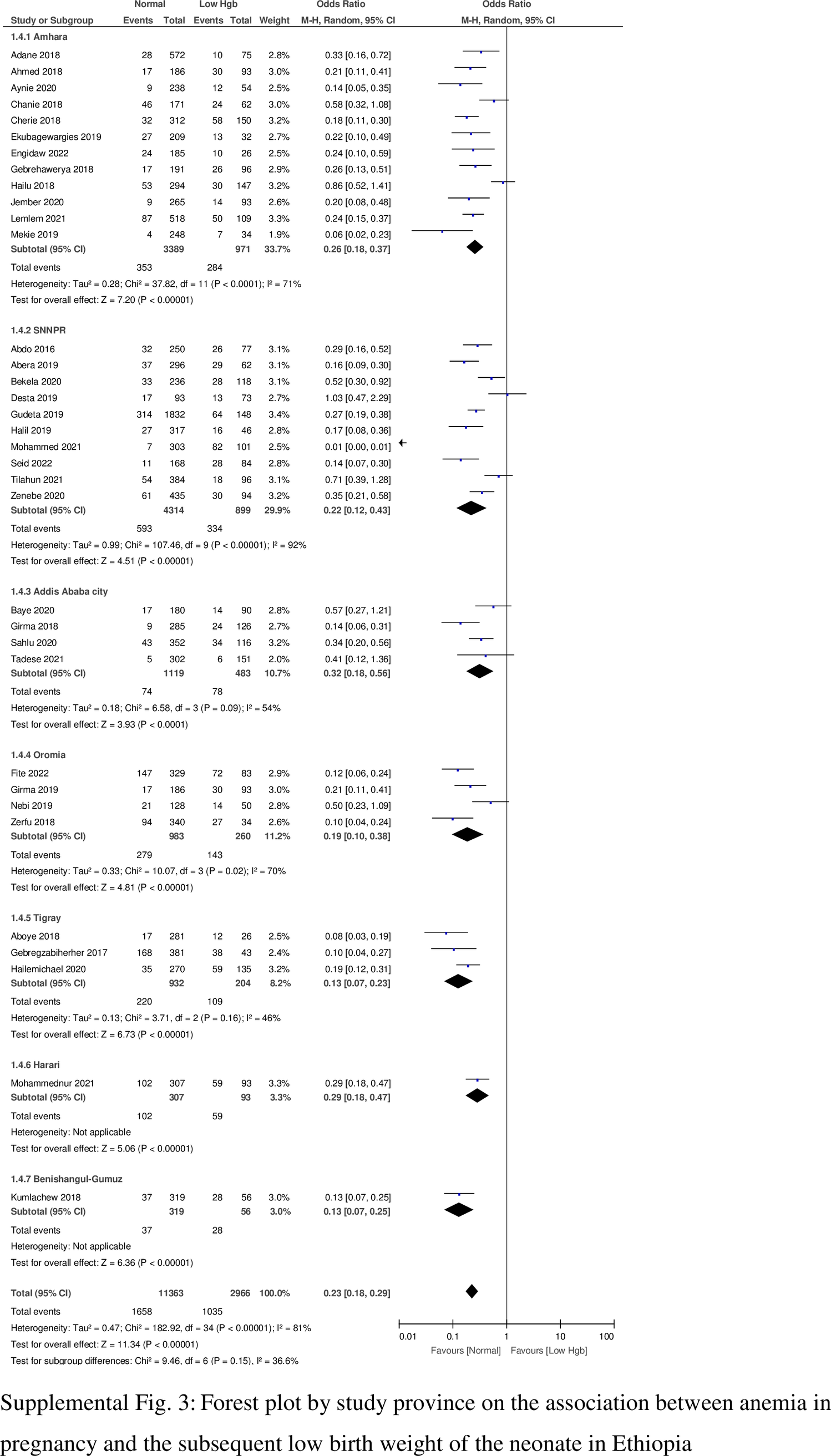

Supplement: S3 Fig — (TIF) [file pone.0310329.s003.tif]

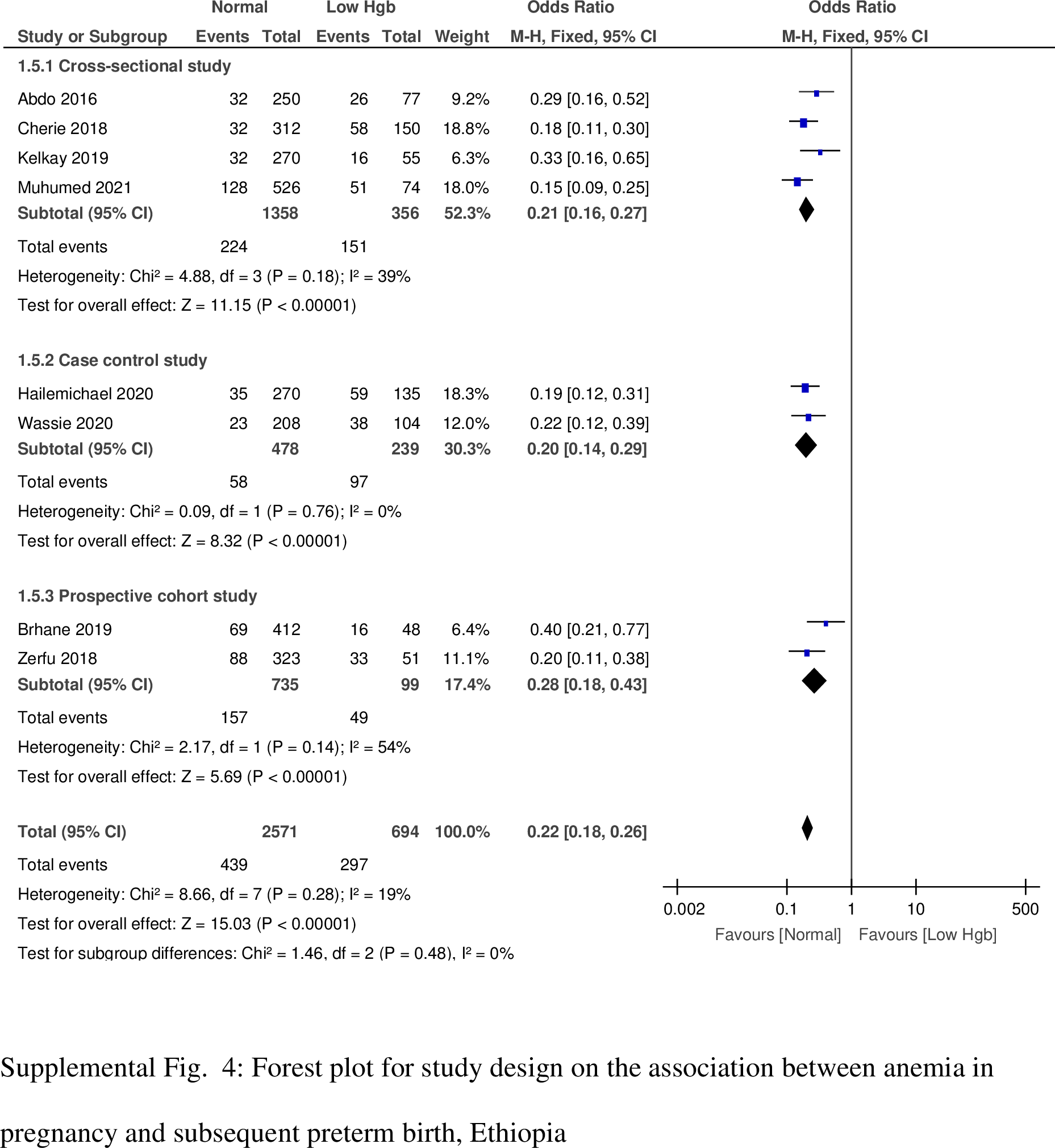

Supplement: S4 Fig — (TIF) [file pone.0310329.s004.tif]

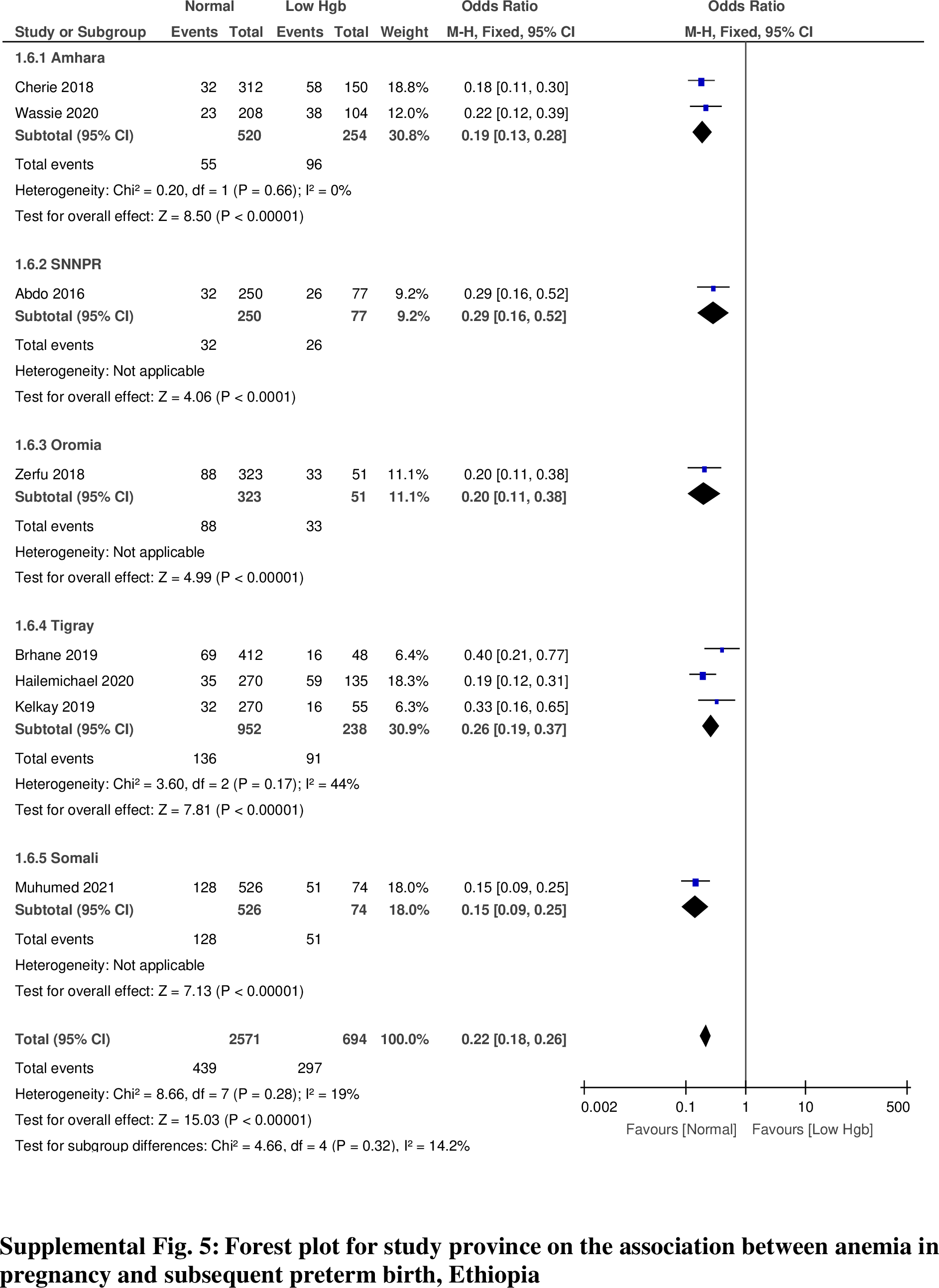

Supplement: S5 Fig — (TIF) [file pone.0310329.s005.tif]

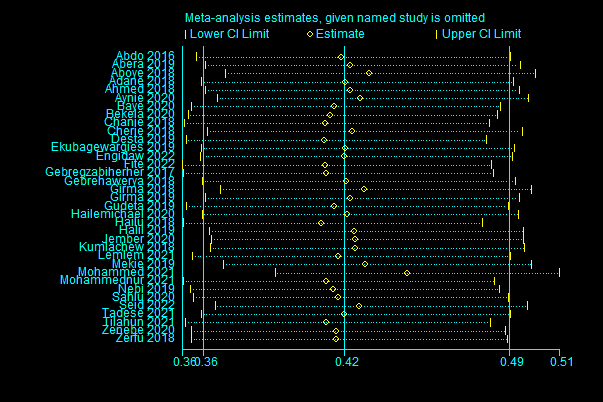

Supplement: S6 Fig — (TIF) [file pone.0310329.s006.tif]

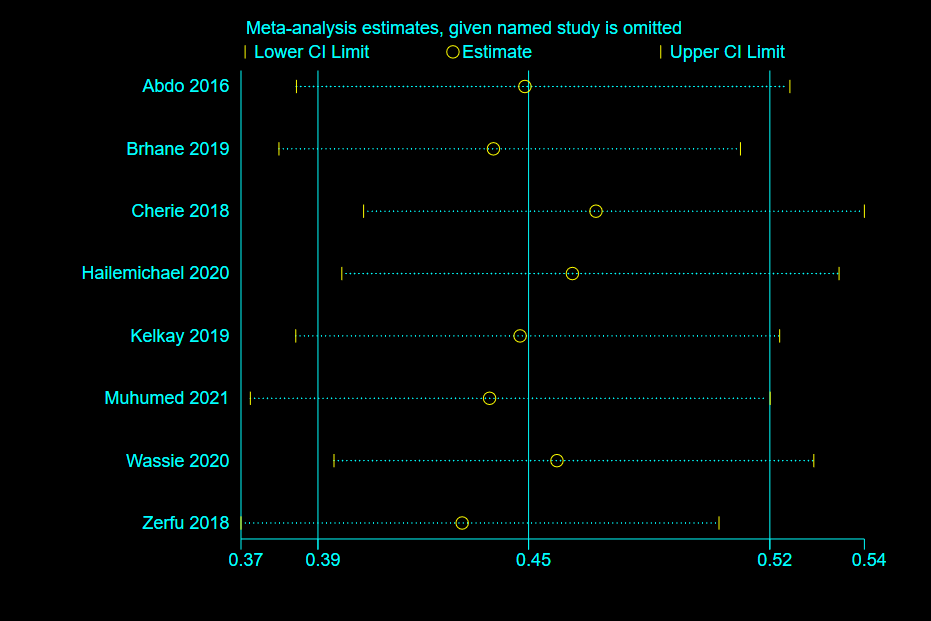

Supplement: S7 Fig — (TIF) [file pone.0310329.s007.tif]
