# Supplementary material for: Association between anemia in pregnancy with low birth weight and preterm birth in Ethiopia: A systematic review and meta-analysis
Source: PLoS One. 2024 Sep 12;19(9):e0310329. doi: 10.1371/journal.pone.0310329 (PMC11392424; doi:10.1371/journal.pone.0310329)
Supplement: S2 Table — (DOCX) [file pone.0310329.s009.docx]

# **Supplemental Table 2: Details of the search strategy**

| 1 | **Search strategy via PubMed, last search on January 30, 2024** | Records retrieved |
| --- | --- | --- |
| S1 | "Pregnant Women"[Mesh] OR "Pregnant Woman" OR "Woman, Pregnant" OR "Women, Pregnant" | 27, 502 |
| S2 | "Pregnancy"[Mesh] OR Pregnancies OR Gestation | 1,214,082 |
| S3 | ("Anemia"[Mesh] OR Anemias) OR ("Hemoglobins"[Mesh] OR Hemoglobin OR Eryhem OR "Ferrous Hemoglobin" OR "Hemoglobin, Ferrous" OR Hematocrit OR Hematocrits) | 495,401 |
| S4 | # S1 OR # S2 AND #S3 | 310,621 |
| S5 | "Infant, Low Birth Weight"[Mesh] OR "Low-Birth-Weight Infant" OR "Infant, Low-Birth-Weight" OR "Infants, Low-Birth-Weight" OR "Low Birth Weight Infant" OR "Low-Birth-Weight Infants" OR "Low Birth Weight" OR "Birth Weight, Low" OR "Birth Weights, Low" OR "Low Birth Weights" | 55,804 |
| S6 | "Birth Weight"[Mesh] OR "Birth Weights" OR "Weight, Birth" OR "Weights, Birth" OR "Birthweight" OR "Birthweights" | 64,629 |
| S7 | # S5 OR # S6 | 102, 676 |
| S8 | "Premature Birth"[Mesh] OR "Birth, Premature" OR "Births, Premature" OR "Premature Births" OR "Preterm Birth" OR "Birth, Preterm" OR "Births, Preterm" OR "Preterm Births" | 36,841 |
| S9 | **# S7 OR # S8** | 128,681 |
| S10 | # S4 AND # S9 | 125,056 |
| S11 | # S10 AND "Ethiopia"[Mesh] | 425 |
| S12 | *Limit S11 to English* | 422 |
| 2 | **Search strategy for Wiley Online Library, last ran on February 02, 2024** |  |
| S1 | "Pregnant Women"[Mesh] OR "Pregnant Woman" OR "Woman, Pregnant" OR "Women, Pregnant" | 983 |
| S2 | "Pregnancy"[Mesh] OR Pregnancies OR Gestation | 45,490 |
| S3 | ("Anemia"[Mesh] OR Anemias) OR ("Hemoglobins"[Mesh] OR Hemoglobin OR Eryhem OR "Ferrous Hemoglobin" OR "Hemoglobin, Ferrous" OR Hematocrit OR Hematocrits) | 33,684 |
| S4 | # S1 OR # S2 AND #S3 | 4,484 |
| S5 | "Infant, Low Birth Weight"[Mesh] OR "Low-Birth-Weight Infant" OR "Infant, Low-Birth-Weight" OR "Infants, Low-Birth-Weight" OR "Low Birth Weight Infant" OR "Low-Birth-Weight Infants" OR "Low Birth Weight" OR "Birth Weight, Low" OR "Birth Weights, Low" OR "Low Birth Weights" | 38 |
| S6 | "Birth Weight"[Mesh] OR "Birth Weights" OR "Weight, Birth" OR "Weights, Birth" OR "Birthweight" OR "Birthweights" | 1012 |
| S7 | S5 OR S6 | 21 |
| S8 | "Premature Birth"[Mesh] OR "Birth, Premature" OR "Births, Premature" OR "Premature Births" OR "Preterm Birth" OR "Birth, Preterm" OR "Births, Preterm" OR "Preterm Births" | 217 |
| S9 | **# S7 OR # S8** | 17 |
| S10 | # S4 AND # S9 | 305 |
| S11 | # S10 AND "Ethiopia"[Mesh] | 295 |
| S12 | # S11 AND English | 294 |
| 3 | **Search strategy for Cochrane Library, last ran on February 01, 2024** |  |
| S1 | "Pregnant Women" AND Anemia OR Hemoglobin AND "Low Birth Weight" OR "birth weight" OR "Preterm Birth" | 384 |
| S2 | "Pregnant Women" AND Anemia OR Hemoglobin AND "Low Birth Weight" OR "birth weight" OR "Preterm Birth" AND Ethiopia | 239 |
| S2 | "Pregnant Women" AND Anemia OR Hemoglobin AND "Low Birth Weight" OR "birth weight" OR "Preterm Birth" AND Ethiopia AND English | 239 |
| 4 | **Google scholar search, on February 02, 2024** |  |
| S1 | "Pregnant Women" AND Anemia OR Hemoglobin AND "Low Birth Weight" OR "birth weight" OR "Preterm Birth" | 18,000 |
| S2 | # S1 AND Ethiopia | 13,300 |
| S3 | # S2 AND English | 5,650 |
