# Supplementary material for: Association between anemia in pregnancy with low birth weight and preterm birth in Ethiopia: A systematic review and meta-analysis
Source: PLoS One. 2024 Sep 12;19(9):e0310329. doi: 10.1371/journal.pone.0310329 (PMC11392424; doi:10.1371/journal.pone.0310329)
Supplement: S3 Table — (DOCX) [file pone.0310329.s010.docx]

Supplemental Table 3: Quality assessment

#### QA-Table 1: Summary score of critical appraisal for methodological quality of included studies using JBI MAStARI for the analytical cross-sectional studies

|  | Criteria for inclusion | Study subject & setting details | Exposure measured | Standard criteria | Confounding factor | Strategies to deal with confounding | Outcome measured in a valid & reliable | Statistical analysis |  |
| --- | --- | --- | --- | --- | --- | --- | --- | --- | --- |
| **Citation** | **Q1** | **Q2** | **Q3** | **Q4** | **Q5** | **Q6** | **Q7** | **Q8** | Score |
| Abera et al., 2019 [1] | Y | Y | Y | Y | Y | Y | Y | Y | 8/8 |
| Aboye et al., 2018 [2] | N | Y | N | Y | Y | Y | N | Y | 5/8 |
| Gebregzabiherher et al., 2017 [3] | Y | Y | Y | Y | Y | Y | Y | Y | 8/8 |
| Halil et al., 2019 [4] | Y | Y | Y | Y | Y | Y | Y | Y | 8/8 |
| Kelkay B,2019 [5] | Y | Y | Y | Y | Y | Y | Y | Y | 8/8 |
| Mekie et al., 2019 [6] | Y | Y | Y | Y | Y | Y | Y | Y | 8/8 |
| Abdo et al., 2016 [7] | N | Y | Y | N | Y | Y | N | Y | 5/8 |
| Cherie & Mebratu, 2017 [8] | Y | Y | Y | Y | Y | Y | Y | Y | 8/8 |
| Jember et al., 2020 [9] | N | Y | Y | Y | Y | Y | Y | Y | 7/8 |
| Aynie et al., 2020 [10] | N | Y | Y | Y | Y | Y | Y | Y | 7/8 |
| Chanie & Dilie,2018 [11] | Y | Y | Y | Y | Y | Y | Y | Y | 8/8 |
| Gudeta et al., 2019 [12] | Y | Y | Y | Y | Y | Y | Y | Y | 8/8 |
| Adane and Dachew, 2018 [13] | Y | Y | Y | Y | Y | Y | N | Y | 7/8 |
| Lemlem et al., 2021[14] | Y | Y | Y | Y | Y | Y | N | Y | 7/8 |
| Ekubagewargies et al., 2019 [15] | Y | Y | Y | Y | Y | Y | Y | Y | 8/8 |
| Muhumed et al., 2021[16] | Y | Y | Y | Y | Y | Y | N | Y | 7/8 |
| Girma & Abebaw, 2018 [17] | Y | Y | Y | Y | Y | Y | Y | Y | 8/8 |
| Kure et al., 2021 [18] | Y | Y | Y | Y | Y | Y | Y | Y | 8/8 |
| Kumlachew et al., 2018 [19] | N | Y | Y | Y | Y | Y | Y | Y | 7/8 |
| Engidaw et al., 2022 [20] | Y | Y | Y | Y | Y | Y | Y | Y | 8/8 |
| % | 66.66 | 100 | 94.44 | 94.44 | 100 | 100 | 72.22 | 100 |  |

NB: % = Percentage; Answer with Y = Yes; N= No

#### QA-Table 2: Summary score of critical appraisal for methodological quality of included studies using JBI MAStARI for the case-control studies

|  | Comparable group | Case & control match | Same criteria for both | Expsure measured in a standard | Exposure measured in the same way | Confounding factors identified | Strategies to deal with confounding | Outcomes assessed | Exposure period long | Statistical analysis |  |
| --- | --- | --- | --- | --- | --- | --- | --- | --- | --- | --- | --- |
| **Citation** | **Q1** | **Q2** | **Q3** | **Q4** | **Q5** | **Q6** | **Q7** | **Q8** | **Q9** | **Q10** | Score |
| Girma et al., 2019 [21] | Y | Y | Y | Y | Y | Y | Y | Y | Y | Y | 10/10 |
| Hailemichael et al., 2020 [22] | Y | Y | Y | Y | Y | Y | Y | Y | Y | Y | 10/10 |
| Hailu & Kebede, 2018 [23] | Y | Y | Y | Y | Y | Y | Y | Y | U | Y | 9/10 |
| Mohammed et al., 2021 [24] | Y | Y | Y | Y | Y | Y | Y | Y | U | Y | 9/10 |
| Mulu et al., 2020 [25] | Y | Y | Y | Y | Y | Y | Y | Y | U | Y | 9/10 |
| Sahlu et al., 2020 [26] | Y | Y | Y | Y | Y | Y | Y | Y | U | Y | 9/10 |
| Ahmed et al., 2018 [27] | Y | Y | Y | Y | Y | Y | Y | Y | U | Y | 9/10 |
| Tilahun & Hailemarium, 2021 [28] | Y | Y | Y | N | Y | Y | Y | N | U | Y | 7/10 |
| Beleke et al., 2020 [29] | Y | Y | Y | Y | Y | Y | Y | Y | U | Y | 9/10 |
| Wassie et al., 2020 [30] | Y | Y | Y | Y | Y | Y | Y | Y | U | Y | 9/10 |
| Gebrehaweriya et al., 2018 [31] | Y | Y | Y | Y | Y | Y | Y | Y | U | Y | 9/10 |
| Nebi et al., 2019 [32] | Y | Y | Y | Y | Y | Y | Y | Y | U | Y | 9/10 |
| Tadese et al., 2021 [33] | Y | Y | Y | Y | Y | Y | Y | Y | U | Y | 9/10 |
| Seid et al., 2022 [34] | Y | Y | Y | Y | Y | Y | Y | Y | U | Y | 9/10 |
| % | 100 | 100 | 100 | 92.31 | 100 | 100 | 100 | 92.31 | 15.38 | 100 |  |

NB: Y= Yes, N= No, U = Unclear

#### QA-Table 3**:** Summary score of critical appraisal for methodological quality of included studies using JBI MAStARI for cohort study

|  | 2 group similar & recruited | Exposure measured to assign people | Exposure measured in a valid & relaible | Confounding factors | Strategy to deal confounding | Groups free of outcome | Outcome measured | Follow up reported | Follow up complete | Strategies to incomplete follow up | Appropriate Statistical analysis |  |
| --- | --- | --- | --- | --- | --- | --- | --- | --- | --- | --- | --- | --- |
| **Citation** | **Q1** | **Q2** | **Q3** | **Q4** | **Q5** | **Q6** | **Q7** | **Q8** | **Q9** | **Q10** | **Q11** | **Score** |
| Desta et al., 2019 [35] | Y | Y | Y | N | Y | Y | Y | U | Y | U | Y | 8/11 |
| Zenebe et al., 2020 [36] | Y | Y | Y | Y | Y | Y | Y | Y | Y | U | Y | 10/11 |
| Brhane et al.,2019[37] | Y | Y | Y | N | Y | Y | U | Y | U | N | Y | 7/11 |
| Zerfu et al., 2018 [38] | Y | Y | Y | N | Y | Y | Y | Y | N | N | Y | 8/11 |
| Fite et al., 2022 [39] | Y | Y | Y | N | Y | Y | Y | Y | N | N | Y | 8/11 |
| % | 100 | 100 | 100 | 20.0 | 100 | 100 | 80.0 | 80.0 | 40.0 | 0.0 | 100 |  |

NB: %, Percentage; Answer with Y= Yes; N= No; U= Unclear

#### QA-Table 4**:** Summary score of critical appraisal for methodological quality of excluded studies using JBI MAStARI for cohort studies

| **Citation** | **Q1** | **Q2** | **Q3** | **Q4** | **Q5** | **Q6** | **Q7** | **Q8** | **Q9** | **Q10** | **Q11** | **Score** |
| --- | --- | --- | --- | --- | --- | --- | --- | --- | --- | --- | --- | --- |
| Tafere et al., 2018 [40] | Y | U | U | N | Y | Y | Y | N | N | N | Y | 5/11 |
| Zerfu et al., 2016 [41] | Y | Y | N | N | N | Y | Y | U | N | N | Y | 5/11 |
| % | 100 | 50.0 | 0.0 | 0.0 | 50.0 | 100 | 100 | 0.0 | 0.0 | 0.0 | 100 |  |

NB: %, Percentage; Answer with Y = Yes; N = No; U = Unclear

1. Abera Z, Ejara D, Gebremedhin S. Nutritional and non-nutritional factors associated with low birth weight in Sawula Town, Gamo Gofa Zone, Southern Ethiopia. BMC Res Notes. 2019;12(1):540. Epub 2019/08/25. doi: 10.1186/s13104-019-4529-0. PubMed PMID: 31443690; PubMed Central PMCID: PMCPMC6708206.

2. Aboye W, Berhe T, Birhane T, Gerensea H. Prevalence and associated factors of low birth weight in Axum town, Tigray, North Ethiopia. BMC Res Notes. 2018;11(1):684. Epub 2018/10/05. doi: 10.1186/s13104-018-3801-z. PubMed PMID: 30285895; PubMed Central PMCID: PMCPMC6167810.

3. Gebregzabiherher Y, Haftu A, Weldemariam S, Gebrehiwet H. The Prevalence and Risk Factors for Low Birth Weight among Term Newborns in Adwa General Hospital, Northern Ethiopia. Obstet Gynecol Int. 2017;2017:2149156. Epub 2017/07/27. doi: 10.1155/2017/2149156. PubMed PMID: 28744313; PubMed Central PMCID: PMCPMC5514323.

4. Halil H, Abdo R, Anshebo A, Hailu A. Low Birth Weight and Risk Factors among Newborns at Nigist Eleni Mohammed Memorial Referral Hospital, Southern Ethiopia: A Cross-Sectional Study. Pediatr & Ther. 2019;9(07):2161-0665.19.

5. Kelkay B, Omer A, Teferi Y, Moges Y. Factors Associated with Singleton Preterm Birth in Shire Suhul General Hospital, Northern Ethiopia, 2018. J Pregnancy. 2019;2019:4629101. Epub 2019/06/18. doi: 10.1155/2019/4629101. PubMed PMID: 31205788; PubMed Central PMCID: PMCPMC6530231.

6. Mekie M, Taklual W. Magnitude of low birth weight and maternal risk factors among women who delivered in Debre Tabor Hospital, Amhara Region, Ethiopia: a facility based cross-sectional study. Ital J Pediatr. 2019;45(1):86. Epub 2019/07/22. doi: 10.1186/s13052-019-0683-1. PubMed PMID: 31324200; PubMed Central PMCID: PMCPMC6642476.

7. Abdo R, Endalemaw T, Tesso F. Prevalence and associated factors of adverse birth outcomes among women attended maternity ward at Negest Elene Mohammed Memorial General Hospital in Hosanna Town, SNNPR, Ethiopia. J Women’s Health Care. 2016;5(4):1000324.

8. Cherie N, Mebratu A. Adverse Birth Out Comes and Associated Factors among Delivered Mothers in Dessie Referral Hospital. North East Ethiopia. 2018:1-6.

9. Jember DA, Menji ZA, Yitayew YA. Low Birth Weight and Associated Factors Among Newborn Babies in Health Institutions in Dessie, Amhara, Ethiopia. J Multidiscip Healthc. 2020;13:1839-48. Epub 2020/12/11. doi: 10.2147/jmdh.S285055. PubMed PMID: 33299321; PubMed Central PMCID: PMCPMC7721311.

10. Aynie AA, Kassa TB, Abie DD. Prevalence of Low Birth Weight and Its Determinants in Bahir Dar City, Amhara Region, North West Ethiopia: Health Facility Based Cross-Sectional Study. Biomedical Statistics and Informatics. 2020;5(1):1.

11. Chanie H, Dilie A. Prevalence of low birth weight and associated factors among women delivered in debre markos referral hospital, East Gojam, Ethiopia, 2017. Prevalence. 2018;53.

12. Gudeta TA, Regassa TM, Gamtesa LC, Lenjebo TL. MAGNITUDE AND FACTORS ASSOCIATED WITH LOW BIRTH WEIGHT AMONG WOMEN DELIVERED IN PUBLIC HOSPITALS OF BENCH MAJI, KEFFA AND SHEKA ZONES SOUTH WEST OF ETHIOPIA, 2018. Ethiopian Journal of Reproductive Health. 2019;11(4):7-.

13. Adane T, Dachew BA. Low birth weight and associated factors among singleton neonates born at Felege Hiwot referral hospital, North West Ethiopia. Afr Health Sci. 2018;18(4):1204-13. Epub 2019/02/16. doi: 10.4314/ahs.v18i4.42. PubMed PMID: 30766587; PubMed Central PMCID: PMCPMC6354871.

14. Lemlem GA, Mezen MK, Atinafu A, Abitew ZA. Maternal factors associated with low birth weight in governmental hospitals of Wollo District, Northeast Ethiopia: a cross sectional study. PAMJ-One Health. 2021;4(18).

15. Ekubagewargies DT, Kassie DG, Takele WW. Maternal HIV infection and preeclampsia increased risk of low birth weight among newborns delivered at University of Gondar specialized referral hospital, Northwest Ethiopia, 2017. Ital J Pediatr. 2019;45(1):7. Epub 2019/01/12. doi: 10.1186/s13052-019-0608-z. PubMed PMID: 30630512; PubMed Central PMCID: PMCPMC6327374.

16. Muhumed II, Kebira JY, Mabalhin MO. Preterm Birth and Associated Factors Among Mothers Who Gave Birth in Fafen Zone Public Hospitals, Somali Regional State, Eastern Ethiopia. Research and Reports in Neonatology. 2021;11:23-33.

17. Girma L. The association between maternal characteristics and low birth weight delivery among neonates delivered in Gandhi Hospital, Addis Ababa: a cross-sectional study. Journal of Family Medicine & Community Health. 2018;5(3).

18. Mohammednur Abdo Komicha GE, Meyrema Abdo, Mohammed Abdurke Kure, Kedir Teji Roba. Magnitude of Low Birth Weight and Associated Factors among Women who gave Birth in Public Hospitals of Harari Regional State, Eastern Ethiopia. Journal of Women’s Health Care. 2021;10 (6):534. doi: 10.35248/2167-0420.21.10.534.

19. Kumlachew W, Tezera N, Endalamaw A. Below normal birth weight in the Northwest part of Ethiopia. BMC Res Notes. 2018;11(1):611. Epub 2018/08/27. doi: 10.1186/s13104-018-3723-9. PubMed PMID: 30144805; PubMed Central PMCID: PMCPMC6109272.

20. Engidaw MT, Eyayu T, Tiruneh T. The effect of maternal anaemia on low birth weight among newborns in Northwest Ethiopia. Scientific Reports. 2022;12(1):15280.

21. Girma S, Fikadu T, Agdew E, Haftu D, Gedamu G, Dewana Z, et al. Factors associated with low birthweight among newborns delivered at public health facilities of Nekemte town, West Ethiopia: a case control study. BMC Pregnancy Childbirth. 2019;19(1):220. Epub 2019/07/04. doi: 10.1186/s12884-019-2372-x. PubMed PMID: 31266469; PubMed Central PMCID: PMCPMC6604438.

22. Hailemichael HT, Debelew GT, Alema HB, Weldu MG, Misgina KH. Determinants of adverse birth outcome in Tigrai region, North Ethiopia: Hospital-based case-control study. BMC Pediatr. 2020;20(1):10. Epub 2020/01/10. doi: 10.1186/s12887-019-1835-6. PubMed PMID: 31914947; PubMed Central PMCID: PMCPMC6947822.

23. Hailu LD, Kebede DL. Determinants of Low Birth Weight among Deliveries at a Referral Hospital in Northern Ethiopia. Biomed Res Int. 2018;2018:8169615. Epub 2018/06/01. doi: 10.1155/2018/8169615. PubMed PMID: 29850570; PubMed Central PMCID: PMCPMC5937567.

24. Mohammed MH, Wabe YA, Ali MM. Determinants of Low Birth Weight Among Newborn Delivered At Public Hospital in Silte Zone, Southern Ethiopia: Case Control Study. 2021.

25. Baye Mulu G, Gebremichael B, Wondwossen Desta K, Adimasu Kebede M, Asmare Aynalem Y, Bimirew Getahun M. Determinants of Low Birth Weight Among Newborns Delivered in Public Hospitals in Addis Ababa, Ethiopia: Case-Control Study. Pediatric Health Med Ther. 2020;11:119-26. Epub 2020/04/11. doi: 10.2147/phmt.S246008. PubMed PMID: 32273790; PubMed Central PMCID: PMCPMC7102875.

26. Sahlu D, Deyessa N, Firdu N, Asfaw S. Food insecurity and other possible factors contributing to low birth weight: A case control study in Addis Ababa, Ethiopia. Asian Pacific Journal of Reproduction. 2020;9(4). doi: 10.4103/2305-0500.288585.

27. Ahmed S, Hassen K, Wakayo T. A health facility based case-control study on determinants of low birth weight in Dassie town, Northeast Ethiopia: the role of nutritional factors. Nutr J. 2018;17(1):103. Epub 2018/11/08. doi: 10.1186/s12937-018-0409-z. PubMed PMID: 30400909; PubMed Central PMCID: PMCPMC6220456.

28. Tilahun T, Hailemariam H. Risk Factors for Low Birth Weight in Sidama Zone Government Hospitals, Southern Ethiopia, A Case-control Study. 2021.

29. Bekela MB, Shimbre MS, Gebabo TF, Geta MB, Tonga AT, Zeleke EA, et al. Determinants of Low Birth Weight among Newborns Delivered at Public Hospitals in Sidama Zone, South Ethiopia: Unmatched Case-Control Study. J Pregnancy. 2020;2020:4675701. Epub 2020/05/01. doi: 10.1155/2020/4675701. PubMed PMID: 32351737; PubMed Central PMCID: PMCPMC7182981.

30. Wassie M, Manaye Y, Abeje G, Tifrie M, Worku G. Determinants of Preterm Birth among Newborns Delivered in Bahir Dar City Public Hospitals, North West Ethiopia. 2020.

31. Gebrehawerya T, Gebreslasie K, Admasu E, Gebremedhin M. Determinants of Low Birth Weight among Mothers Who Gave Birth in Debremarkos Referral Hospital, Debremarkos Town, East Gojam, Amhara Region, Ethiopia. Neonatal and Pediatric Medicine. 2018;04(01). doi: 10.4172/2572-4983.1000145.

32. Nebi NO, Chaka TE, Abebe TW. Risk Factors for Low Birth Weight among Neonates Delivered in Public Health Facilities in Adama town, Oromia Regional State, Ethiopia. 2019.

33. Tadese M, Minhaji AS, Mengist CT, Kasahun F, Mulu GB. Determinants of low birth weight among newborns delivered at Tirunesh Beijing General Hospital, Addis Ababa, Ethiopia: a case-control study. BMC Pregnancy and Childbirth. 2021;21(1):1-9.

34. Seid S, Wondafrash B, Gali N, Ali A, Mohammed B, Kedir S. Determinants of Low Birth Weight Among Newborns Delivered in Silte Zone Public Health Facilities, Southern Ethiopia: A Case-Control Study. Research and Reports in Neonatology. 2022:19-29.

35. Desta M, Tadese M, Kassie B, Gedefaw M. Determinants and adverse perinatal outcomes of low birth weight newborns delivered in Hawassa University Comprehensive Specialized Hospital, Ethiopia: a cohort study. BMC Res Notes. 2019;12(1):118. Epub 2019/03/06. doi: 10.1186/s13104-019-4155-x. PubMed PMID: 30832723; PubMed Central PMCID: PMCPMC6399950.

36. Zenebe A, Eshetu B, Gebremedhin S. Association between maternal HIV infection and birthweight in a tertiary hospital in southern Ethiopia: retrospective cohort study. Ital J Pediatr. 2020;46(1):70. Epub 2020/05/26. doi: 10.1186/s13052-020-00834-3. PubMed PMID: 32448252; PubMed Central PMCID: PMCPMC7247191.

37. Brhane M, Hagos B, Abrha MW, Weldearegay HG. Does short inter-pregnancy interval predicts the risk of preterm birth in Northern Ethiopia? BMC Res Notes. 2019;12(1):405. Epub 2019/07/17. doi: 10.1186/s13104-019-4439-1. PubMed PMID: 31307529; PubMed Central PMCID: PMCPMC6631733.

38. Zerfu TA, Pinto E, Baye K. Consumption of dairy, fruits and dark green leafy vegetables is associated with lower risk of adverse pregnancy outcomes (APO): a prospective cohort study in rural Ethiopia. Nutr Diabetes. 2018;8(1):52. Epub 2018/09/22. doi: 10.1038/s41387-018-0060-y. PubMed PMID: 30237477; PubMed Central PMCID: PMCPMC6148027 involvement in the study as an advisor. ETHICAL CONSIDERATION: After a detailed and face to face explanation of the purpose and methods of the study to all women, informed verbal consent was obtained from in the presence of local administrators. All the study procedures were performed in accordance with the Helsinki Declaration. The study protocol was approved by the institutional review boards of the College of Natural Sciences, Addis Ababa University and ethics review committee of Oromia Regional Health Bureau. CONFLICT OF INTEREST: The authors declare that they have no conflict of interest.

39. Fite MB, Tura AK, Yadeta TA, Oljira L, Roba KT. Prevalence, predictors of low birth weight and its association with maternal iron status using serum ferritin concentration in rural Eastern Ethiopia: a prospective cohort study. BMC nutrition. 2022;8(1):1-10.

40. Tafere TE, Afework MF, Yalew AW. Providers adherence to essential contents of antenatal care services increases birth weight in Bahir Dar City Administration, north West Ethiopia: a prospective follow up study. Reprod Health. 2018;15(1):163. Epub 2018/10/01. doi: 10.1186/s12978-018-0610-8. PubMed PMID: 30268132; PubMed Central PMCID: PMCPMC6162936.

41. Zerfu TA, Umeta M, Baye K. Dietary diversity during pregnancy is associated with reduced risk of maternal anemia, preterm delivery, and low birth weight in a prospective cohort study in rural Ethiopia. Am J Clin Nutr. 2016;103(6):1482-8. Epub 2016/05/14. doi: 10.3945/ajcn.115.116798. PubMed PMID: 27169832.
