# Supplementary material for: Association between anemia in pregnancy with low birth weight and preterm birth in Ethiopia: A systematic review and meta-analysis
Source: PLoS One. 2024 Sep 12;19(9):e0310329. doi: 10.1371/journal.pone.0310329 (PMC11392424; doi:10.1371/journal.pone.0310329)
Supplement: S4 Table — (DOCX) [file pone.0310329.s011.docx]

Supplemental Table 4: List and reasons of ineligible studies following full-text review

| No |  | Reason for exclusions |
| --- | --- | --- |
| 1 | Tafere TE, Afework MF, Yalew AW. Providers adherence to essential contents of antenatal care services increases birth weight in Bahir Dar City Administration, north West Ethiopia: a prospective follow up study. Reproductive health. 2018 Dec;15(1):1-8. | The study was deemed to have insufficient information concerning exposure of interest. |
| 2 | Zerfu TA, Umeta M, Baye K. Dietary diversity during pregnancy is associated with reduced risk of maternal anemia, preterm delivery, and low birth weight in a prospective cohort study in rural Ethiopia. The American journal of clinical nutrition. 2016 Jun 1;103(6):1482-8. | There was not sufficient information on exposure of interest and outcomes. |
| 3 | Birtukan Alemu, Dawd Gashu. Association of maternal anthropometry, hemoglobin and serum zinc concentration during pregnancy with birth weight. Early Hum Dev. 2020 Mar;142:104949. doi: 10.1016/j.earlhumdev.2019.104949. Epub 2020 Jan 7. | There was not sufficient information on exposure of interest |
| 4 | [Alemu Basazin Mingude](https://pubmed.ncbi.nlm.nih.gov/?term=Mingude+AB&cauthor_id=32782793) , [Woiynshet Gebretsadik](https://pubmed.ncbi.nlm.nih.gov/?term=Gebretsadik+W&cauthor_id=32782793),  [Dresilgn Misker](https://pubmed.ncbi.nlm.nih.gov/?term=Misker+D&cauthor_id=32782793) , [Gashaw Garedew Woldeamanuel](https://pubmed.ncbi.nlm.nih.gov/?term=Woldeamanuel+GG&cauthor_id=32782793). Determinants of low birth weight among live birth newborns delivered at public hospitals in Gamo Gofa Zone, SouthEthiopia: Unmatched case control study. SAGE Open Medicine. 2020, Volume 8: 1–8. | There was not sufficient information on exposure of interest and outcomes. |
| 5 | Gashaw Garedew Woldeamanuel , Teshome Gensa Geta, Tesfaye Petros Mohammed, Mulualem Belachew Shuba, and Temesgen Abera Bafa. Effect of nutritional status of pregnant women on birth weight of newborns at Butajira Referral Hospital, Butajira, Ethiopia, SAGE Open Medicine, Volume 7: 1 –7, 2019. | There was not sufficient information on exposure of interest and outcomes. |
| 6 | Animut Alebel, Fasil Wagnew, Cheru Tesema, Alemu Gebrie, Daniel Bekele Ketema, Getnet Asmare, and Getiye Dejenu Kibret. Factors associated with low birth weight at Debre Markos Referral Hospital, Northwest Ethiopia: a hospital based cross-sectional study, BMC Research Notes , (2019) 12:105. | There was not sufficient information on exposure of interest |
